# Systematic Clustering of Transcription Start Site Landscapes

**DOI:** 10.1371/journal.pone.0023409

**Published:** 2011-08-24

**Authors:** Xiaobei Zhao, Eivind Valen, Brian J. Parker, Albin Sandelin

**Affiliations:** Department of Biology and Biotech Research and Innovation Centre, The Bioinformatics Centre, Copenhagen University, Copenhagen, Denmark; University College London, United Kingdom

## Abstract

Genome-wide, high-throughput methods for transcription start site (TSS) detection have shown that most promoters have an array of neighboring TSSs where some are used more than others, forming a distribution of initiation propensities. TSS distributions (TSSDs) vary widely between promoters and earlier studies have shown that the TSSDs have biological implications in both regulation and function. However, no systematic study has been made to explore how many types of TSSDs and by extension core promoters exist and to understand which biological features distinguish them. In this study, we developed a new non-parametric dissimilarity measure and clustering approach to explore the similarities and stabilities of clusters of TSSDs. Previous studies have used arbitrary thresholds to arrive at two general classes: broad and sharp. We demonstrated that in addition to the previous broad/sharp dichotomy an additional category of promoters exists. Unlike typical TATA-driven sharp TSSDs where the TSS position can vary a few nucleotides, in this category virtually all TSSs originate from the same genomic position. These promoters lack epigenetic signatures of typical mRNA promoters and a substantial subset of them are mapping upstream of ribosomal protein pseudogenes. We present evidence that these are likely mapping errors, which have confounded earlier analyses, due to the high similarity of ribosomal gene promoters in combination with known G addition bias in the CAGE libraries. Thus, previous two-class separations of promoter based on TSS distributions are motivated, but the ultra-sharp TSS distributions will confound downstream analyses if not removed.

## Introduction

The recruitment of the pre-initiation complex (PIC) to the transcription start site (TSS) is a complex interplay of many factors, including binding of transcription factors and epigenetic signals such as nucleosome occupancy and modification of histone tails [1]. Since the TSS can be regarded as the focal point in the activation of transcription, much effort has been invested in experimental and computational methods to identify TSSs and core promoters. The completion of several genomes of higher eukaryotes has prompted the development of accurate genome-wide methods based on capturing capped transcripts and sequencing the first 20–30 nt from the 5′ end of these using high-throughput DNA sequencers. Examples of these include Cap Analysis of Gene Expression (CAGE) [2], massively parallel Paired End Tag (PET)-tagging [3] and Oligocapping [4]. These 20–30 nucleotide (nt) long tags are then mapped back to the genome to indicate the location of TSSs, with nucleotide-level resolution [5]. Importantly, the number of tags mapping to a certain genomic region can be regarded as a measure of the amount of transcription initiation from this region, and these techniques can also be used to identify promoters that are only used in certain tissues [6].

These methods have been used to provide the scientific community with promoter maps over multiple genomes and tissues [2], [3], [7], dissect core promoter architecture on nucleotide level [2], [8], [9], [10], [11], [12], explore alternative promoter selection and transcription initiation diversity [6], [13], [14], [15], [16], unravel promoter-based regulatory networks [17], [18], assess evolutionary constraints of regulation [19], [20] and more.

On a more fundamental level, the methods have shown that most core promoters have an array of initiation sites that are used with different intensities, instead of a single initiation site governed by a TATA-box [10]. Therefore, the initiation sites of a promoter are better described as a TSS distribution (TSSD), where some TSSs are used more than others. TSSDs are generally conserved over species and tissues [10], although notable exceptions exist where the distribution shifts between cells [13], [19]. Moreover, within a promoter, the initiation site propensity can be predicted by the surrounding DNA sequence [21]. Carninci *et al.* [10] showed that the shape of the TSSDs in human and mouse is correlated to both sequence content and tissue expression. The study tried to make sense of this phenomenon by dividing the core promoters into four arbitrarily defined classes based on their TSSDs, using a simple rule-based classification system (broad, multi-modal, broad-with-peak, and sharp promoters), and then analyzed the features of the four classes. Subsequent studies have often reduced these classes to simple “broad” and “sharp”. Similar rule-based systems have been proposed for *Drosophila melanogaster* promoters [16].

In human and mouse, promoters with many start sites (“broad promoters”) are generally less conserved, more ubiquitously expressed, CpG-rich and TATA-depleted compared to the promoters with one or a few densely aggregated TSSs (“sharp promoters”) (reviewed in [1], [22], [23]) and these promoters also use distinct strategies in nucleosome organization and chromatin structure [24]. These findings complement studies splitting promoters based on normalized CG content [25] or according to the surrounding epigenetic signals at TSSs of the promoters [26],and make it clear that the division of core promoters based on their TSS distribution is meaningful since different modes of gene regulations are used in the different classes, which will confound downstream analysis if not separated.

A potential problem with all of these studies is that the TSSD classes are arbitrarily defined: we do not know whether it is the most relevant to assume there are two, four or even more subclasses of promoters. As noted in [22], a relevant promoter classification is important for the experimental and computational detection of regulatory mechanism including *cis*-elements and *trans*-acting factors, as much of the noise is due to indiscriminate mixing of classes. This is the goal of the current study: we extend previous studies further by using a unsupervised learning framework to explore different TSSDs in mammalian genomes, in order to i) ascertain if the classification systems of precious studies are justified, ii) find out if further subclasses exist, and what sets these apart from a biological viewpoint.

## Results

In this section we will describe a quantitative metric to measure how dissimilar a TSSD is from another, a two-level clustering exploration using this metric and finally an analysis of the three promoter classes that emerged using both sequence and epigenetic features.

### Representation of core promoters by TSSDs

The activity of a core promoter can be described as a distribution of TSS usage within a small genomic region. In this study, we focus on the distribution of CAGE tags since this is the largest data set to date from multiple tissues: in particular, we use the FANTOM3 CAGE data from 22 tissues in mouse, provided by Carninci *et al.* [10]. Besides being a large and diverse set this also gives us the opportunity to directly compare our results with the four-class grouping introduced in that study. As discussed below, we also use other CAGE datasets from other tissues and species in order to generalize our findings (Table S1).

In Carninci *et al.* [10], nearby TSSs on the same strand were clustered into “tag clusters” based on tags overlapping with at least one nucleotide, and the distribution of TSSs within these genomic clusters were assessed for clusters having at least 100 tags. In this study, we used the same clusters, with a few modifications to reduce “tiling” artifacts in the borders of the distributions (see Methods). In total, our primary data set consists of 7,752 TSSDs containing 5,463,328 uniquely mapped CAGE tags.

We will avoid the term “tag cluster” in the rest of the text and instead refer to these collections of tags as TSS distributions (TSSDs), since we will later cluster TSSDs and form larger aggregates (in other words clusters of clusters). For clarity, in the rest of the text we will refer to *clustering* as a process or an assignment of a set of TSSDs into subsets according to its common use in cluster analysis. We also refer to *clusters* as the subsets generated by a clustering and refer to *partition* as a collection of all subsets in one clustering.

### Measuring the dissimilarity between two TSSDs

In order to define clusters of TSSDs one first needs a metric that defines how similar or dissimilar TSSDs are to each other. This should be robust and intuitive in order to give meaningful results. In our case, it is more likely that the shape of the TSSD rather than the magnitude of expression (which is influenced by many external factors) is indicative of core promoter organization and we therefore normalized the CAGE tag count of each TSSD to sum to one.

We experimented with various measures (See Methods) and in the end chose a non-parametric distance measure based on Minimum Difference of Pair Assignments (MDPA) [27], which is similar to the Earth Mover's Distance (EMD) [28] and is a true metric. As TSSDs can have different lengths, we modified the method slightly (See Methods) and called our modified distance measure “Generalized Minimum distance of distributions” (GM-distance; see Methods). An intuitive description of GM-distance is that it measures the dissimilarity between two TSSDs *A* and *B* by counting the number of one-nucleotide “shifts” of tags that have to be performed within distribution *A* to make it into distribution *B*. The more similar two TSSDs are, the less moving steps are needed and the less distance is between them. Given this method, we calculated the dissimilarities between all pairs of TSSDs, forming a 7752×7752 dissimilarity matrix.

### Cluster analysis indicates a gradient of TSS distributions

**Figure 1:**
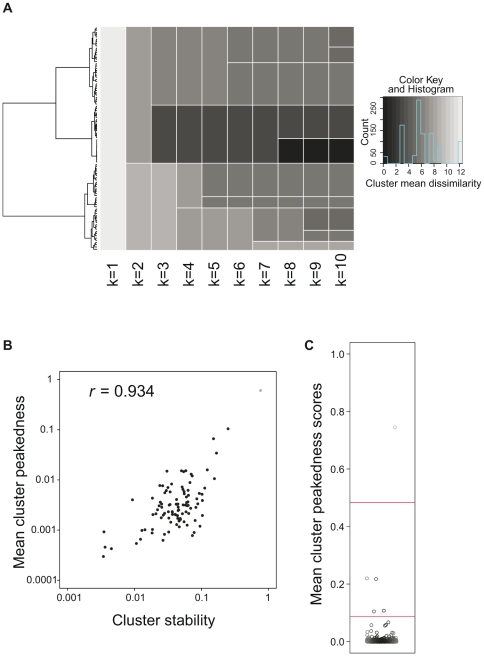
Clustering of transcription start site distributions (TSSDs). (A) Example of the heterogeneity of TSSDs in clustering based on dissimilarity alone. We clustered 100 randomly sampled TSSDs using hierarchical clustering, shown as a dendrogram on the left. The heatmap represent the different partitions that can be produced by placing a cut-line vertically in the dendrogram at various places: the second column shows the two-cluster partition (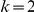), the next the three cluster partition (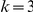), etc. The color intensity indicates the mean dissimilarity between all the TSSDs within one cluster (darker means higher homogeneity). Note that most clusters are inhomogeneous when *k* is low: clusters with high homogeneity only emerge when moving the cutline closer to the leaves. (B) Correlation between the mean cluster peakedness and the cluster stability. The scatter plot compares the cluster stability scores resulting from the bootstrap resampling to the intra-cluster peakedness scores. *R* denotes the Pearson's correlation coefficient of the scores (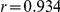). (C) Distribution of intra-cluster peakedness scores of 500 TSSD clusters generated by hierarchical clustering. The Y-axis shows the intra-cluster peakedness scores. The red lines indicate offsets for defining three larger clusters using *k*-means. Each box represents one TSSD cluster.

Using the dissimilarity matrix defined above, we first explored the TSSD relationships by using hierarchical clustering (see Methods), motivated by that the method in itself does not define a specific number of clusters. A standard way to obtain clusters from a dendrogram produced by hierarchical clustering is to “cut” the dendrogram at a defined depth where a cut line close to the root will generate relatively few large clusters and conversely a cut close to the leaves of the dendrogram will give many smaller clusters. We explored the consistency of the clustering by first imposing cuts that gave 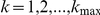 clusters, where 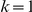 represents the original data set without partitioning. The choice of *k* is arbitrary depending on how deep we want to investigate into the dendrogram structure. For explorative purposes, we set *k_max_* to 500 in order to include a large number of possible partitions while still maintaining reasonable sizes of the clusters. We then measured the intra-cluster (mean) dissimilarity of the TSSDs within all produced clusters. The intra-cluster dissimilarity is computed by averaging all the pairwise dissimilarities of the TSSDs in that cluster. Lower intra-cluster dissimilarity indicates increased homogeneity of the cluster. We find that sub-clusters with high homogeneity only emerge from previously defined clusters at a late stage when we increase the number of clusters to large values; in other words the boundaries between most classes are not very sharp and there are no immediate outliers that emerge early. As an illustration of this, we constructed a dendrogram based on 100 randomly sampled TSSDs from the primary data set (Figure 1A). Ten partitions were produced by cutting this dendrogram into 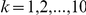 clusters. In this example, homogenous clusters only occur when *k* is larger than 8, and these clusters can be divided to even more homogenous sub-clusters (not shown). This indicates that overall, the data set is highly heterogeneous, and one needs to place the cut line far from the root to identify partitions with more homogeneous clusters.

We can then select different values of *k* and deduce what number of groups that fits the data best. One common method to assess the performance of a clustering model is to measure either the explained variance or the residual variance of the model. Since the total variance is constant for a given dataset, models with small total residual variance (close to 0) or large total explained variance (close to 100%) is optimal in explaining most of the variation (and thus the heterogeneity) in the data. Figure S1A shows the percentage of variance in the data that is explained as a function of the number of clusters (*k*).

With the increasing number of clusters, the explained variance tends to increase, whereas the additional variance explained in each step usually decreases. We find around 50% of the total variance is explained by the two-cluster solution, and approximately 80% is explained by the ten-cluster solution. To determine the optimum number of clusters, an elbow criterion is typically employed - the optimum number of clusters is taken where a sudden change occurs in the graphical curve. Figure S1A indicates that the elbow [29] can lie anywhere between 2 and 10 clusters. Thus, it is hard to define the number of clusters based on the dissimilarity alone. This characteristic is not specific to the clustering method: we observe the same heterogeneity if using *k*-medoids clustering instead of hierarchical clustering (see Text S1).

To address this, we next assessed how statistically robust the proposed groups are: will the same groups be discovered if we only have a randomly resampled dataset? Using a bootstrap method [30], we resampled the 7752 TSSDs with replacement from the original data set and performed the same hierarchical and *k*-medoids clustering on the new data as described above, producing 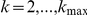 partitions for each resampling. For each *k*, we calculated the weighted mean of the cluster mean Jaccard coefficients, which will correspond to the overall stability of the clustering (see Methods).

As shown in Table S2, the cluster stability is high for 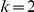 and it gradually decreases as *k* is increased. This is unsurprising because for a larger 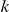 there are a greater number of possible solutions that are close to the global optimum, and therefore the stability remains moderate. The stability does not decrease monotonically as 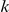 increases, with some maxima slightly peaked locally in stability values such as for 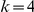 by hierarchical clustering and 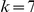 by *k*-medoids clustering (see Methods). However, the stability values show some variation between the different clustering methods, so it is unclear whether these maxima are robust.

In summary, the dataset has weak clustering tendencies using this dissimilarity measure alone: this is corroborated both by the fact that no clear groups are standing out near the root of the clustering tree and there is no particular set of clusters that is more stable than the others. Since our dissimilarity measure is measuring how similar TSSDs are in terms of number of tags that have to be moved, this indicates that the dataset consists of a shallow gradient of distributions. At the same time, the dissimilarity method might not detect some of the most relevant features in the data for the problem at hand: for instance, two TSSDs that each have two major peaks but where these peaks are spaced differently would be considered dissimilar with our measure, since an entire peak would have to be “moved”. We will revisit the possible reasons for the large variation in TSSDs below.

To confirm that the lack of clustering is not a consequence of the origin of the data, the stability analysis was also carried out on two mouse CAGE libraries from different tissues and also two human CAGE data sets (Table S3A–B). The cluster stability is once again high for 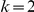 and slowly decreases as *k* is increased, suggesting that our cluster stability arguments also apply to data sets from other tissues and species.

### Two-level clustering categorizes TSSDs into three primary types: scattered, dense and ultra-dense TSSDs

In the process of investigating the stability above, we made two observations: firstly, some highly stable child clusters will not emerge from their parent cluster until the total number of clusters (*k*) is high (or, equivalently, when the cut line is close to the leaves of the dendrogram). Secondly, we noticed that the most stable clusters are characterized by TSSDs where most TSSs originate from a few nucleotides, which makes intuitive sense. We then examined whether this correlation was true for the entire dataset by first calculating a “peakedness score” (see Methods) for each TSSD.

At first, we tried to use the peakedness scores as a replacement for our distance measure described above, however, this did not produce meaningful results (data not shown). This is likely due to that the peakedness score in itself only captures the weight of the highest peak and the broadness of the distribution, but ignores the actual shape of the distribution (which the original distance measure captures). For instance, two TSSDs may have the same peakedness scores but overall different distributions. Therefore we considered combining the peakedness measure with previous clustering based on the shapes of the distribution.

We introduced the term of “intra-cluster” (mean) peakedness and started with the comparison of the intra-cluster peakedness of TSSDs within a cluster with its stability defined by the mean cluster Jaccard coefficients (see Methods), and concluded that there is a strong and significant correlation between these statistics given 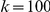 (Figure 1B) (Pearson correlation coefficient *r* = 0.934, *P*<2.2e-16, cor.test in R). Conclusively, the intra-cluster peakedness is a reasonable approximation of the intra-cluster stability. More important, this stability approximation captures both the peakedness and the structure of the hierarchical clustering, by averaging the individual peakedness scores in a cluster-wise assignment. Using this approximation is also sensible in terms of computational cost since the stability calculations by bootstrapping are computationally expensive.

Since clustering based on dissimilarity alone could not identify highly stable clusters without increasing *k* to large values, which produces many clusters and some of them have very few TSSDs, we reasoned that including the peakedness of the proposed clusters as an additional feature would identify the most stable clusters at a early stage during cluster analysis and generate fewer clusters with more TSSDs.

To achieve this, we first conducted a hierarchical clustering (1st-level clustering) based on the dissimilarity measure by GM-distance, and obtained one *500*-cluster partition given a cutoff of 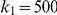 as described above. We calculated 500 intra-cluster (mean) peakedness scores of all clusters in this partition. Plotting the distribution of these intra-cluster peakedness scores show a clear separation of TSSD clusters where most have low peakedness (Figure 1C). To separate these from each other in a systematic way, we used *k*-means to divide the TSSD clusters based on their intra-cluster peakedness scores (a second-level clustering), *i.e.* aggregating the 1st-level clusters based on their cluster peakedness measure and producing 2nd-level clusters. We tested what number of 2nd-level clusters (*k*_2_) that explained the variance in peakedness best. We found that when setting 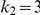, 99% of the variance could be explained, while larger values of 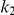 gave no substantial improvement (Figure S2). Therefore the clusters from the 1st-level clustering are aggregated further into three 2nd-level clusters: composed of 1, 4, and 495 1st-level clusters, respectively (Figure 1C).

**Figure 2:**
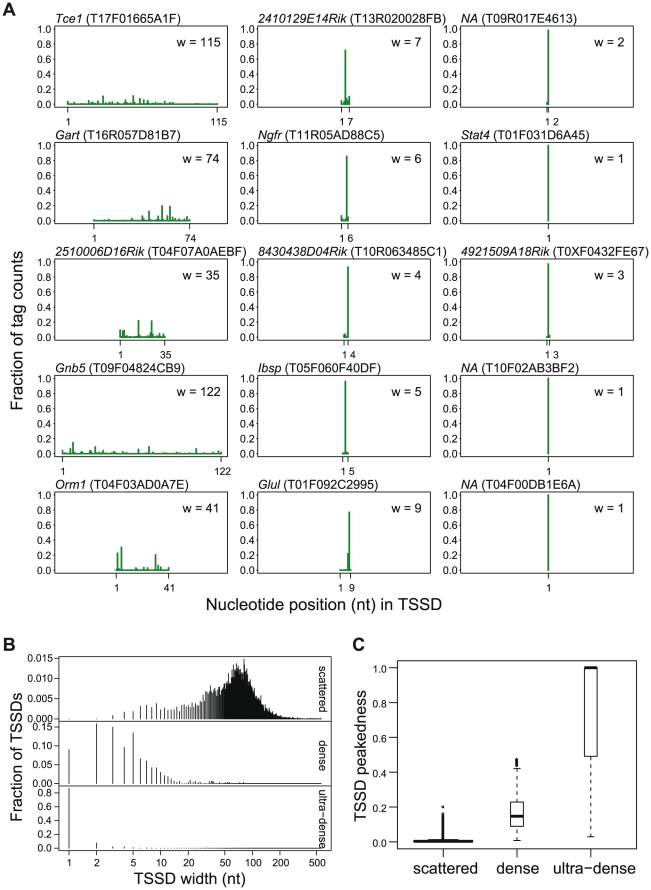
Properties of “scattered”, “dense” and “ultra-dense” TSSD clusters. (A) Examples of individual TSSDs of respective class: scattered TSSDs(left column), dense TSSDs (middle column) and ultra-dense TSSDS (right column) . The X-axis shows the relative genomic position with the 5′ end of the distribution is placed at coordinate 1. The Y-axis shows the fraction of the tags. The text above each distribution gives gene names or transcriptional unit identifiers of the TSSD in FANTOM3 database and the TSSD identifier is in brackets. The inset gives the width of the TSSD. (B) Distribution of the TSSD widths. The width distribution characterizes how dense the TSSDs are. Scattered, dense and ultra-dense TSSDs are in the top, middle, bottom panels, respectively. The X-axis shows the width of the TSSDs in unit of nt. Scattered TSSDs are mainly in the range from 20 nt to 200 nt; dense TSSDs are generally less than 20 nt long; ultra-dense TSSDs are in most cases 1 nt wide. (C) Box-plots showing the distribution of peakedness scores of individual TSSDs. Scattered, dense and ultra-dense TSSDs are in the left, middle, right boxes, respectively.

The analysis directly results in two stable clusters, i) “dense TSSDs” where many, but not all TSSs are co-localized (334 TSSDs, 4%) and ii) “ultra-dense TSSDs” where virtually almost all TSSs originate from the same nucleotide (323 TSSDs, 4%), and finally one cluster which is large but has low peakedness and low stability, identifying TSSDs with scattered TSS distributions that, as discussed above, are too diverse to be easily clustered to smaller clusters. The last cluster is dominating the data set, covering 91% of the total number of TSSDs (Table S4). Properties of the classes and examples of TSSd of each alss are shown in Figure 2. We made a further study on possible ways to sub-cluster the scattered set below.

We compared these proposed clusters to the original four classes suggested by Carninci *et al.* [10] (Table 1). As expected, more than 90% of dense and ultra-dense TSSDs were labeled “single peak (SP)” under Carninci's four-class scheme; while the scattered TSSDs were labeled of BR, MU, PB and SP with slight preference to BR promoters. However, many of the original SP–class promoters are labeled as “scattered”, probably reflecting that some of these have substantial tiling effects (where a substantial number of tags are overlapping each other by a few nucleotides, thus creating a wide distribution) that were reduced but not completely removed by our pre-filters, as shown in Figure S3. In Carninci *et al* [10] this problem was sidestepped by the rules of their hierarchical assignment strategy – single peaks assignment was based on percentiles within a sliding 4-nt window, which is not highly affected by tiling effects.

**Table 1:** Comparison of the three-class TSSD with Carninci's four-class scheme.

Carninci's class	scattered (%)	dense (%)	ultra-dense (%)	Total
**BR**	2647 (37.3%)	0 (0.0%)	0 (0.0%)	2647
**MU**	1473 (20.8%)	2 (0.6%)	0 (0.0%)	1475
**PB**	1758 (24.8%)	2 (0.6%)	0 (0.0%)	1760
**SP**	1215 (17.1%)	329 (98.5%)	293 (90.7%)	1837
**-**	2 (0.0%)	1 (0.3%)	30 (9.3%)	33
**Total**	7095 (100.0%)	334 (100.0%)	323 (100.0%)	7752

Comparison of TSSD clusters identified by two-level clustering with Carninci's four-class scheme (Carninci *et al.* [32]). TSSDs with missing labels in Carninci's scheme are denoted by “-”.

### Biological features of different TSSD clusters

Next, we wanted to see what biological features set these different TSSDs and associated promoter regions apart. In particular, since we have a considerable overlap with the four-class classification by Carninci *et al.* [10] as discussed above, we wanted to see if the correlations reported in that study holds in the new classification.

#### Sequence patterns, tissue expression and evolutionary sequence conservation

**Figure 3 pone-0023409-g003:**
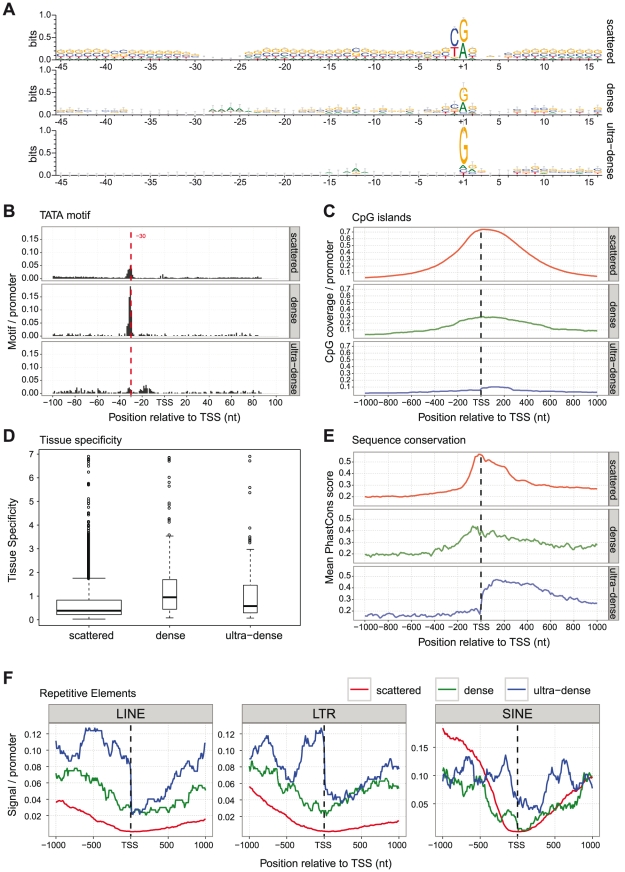
Sequence and expression features of “scattered”, “dense” and “ultra-dense” TSSD classes. For each class, the TSSDs are aligned at their dominant peaks (labeled “TSS” at X-axis). (A) Sequence properties of promoters divided by TSSD class. Sequence logos [68] of the DNA sequence of the TSSDs aligned at the dominant TSS. The x-axis shows the relative genomic positions, +1 indicates TSS. The y-axis shows the information content measured in bits. (B) TATA-box density of promoters divided by TSSD class. The count of predicted TATA sites flanking the dominant TSS (+/−100 nt) of the TSSDs. The X-axis shows the positions of the first T of the TATA site relative to the dominant TSS in the +−100 region; the Y-axis shows the number of predicted sites per TSSD. Note that the absolute frequencies of predicted sites are strongly dependent on the cutoffs, but the relative difference between different TSSDs are not cutoff-dependent. TATA sites are strongly over-represented at around −32 nt in the dense group (middle panel) but are less defined in the scattered group (top panel). The ultra-dense group (bottom panel) shows a small TATA signal located at either −32 nt or around −20 nt. (C) CpG island coverage of promoters divided by TSSD class. The coverage of CpG islands is illustrated in the flanking region (+/−1000 nt) around the TSSs. The X-axis shows the genomic position relative to the TSSs; the Y-axis shows the number of nucleotides covered by a CpG islands/TSSD. (D) Tissue specificity of TSSD classes. The box-plots show the distribution of the overall tissue specificity, given the class of the TSSD, calculated as the KullbacK-Leibler divergence. The smaller the distance is, the lower is the tissue specificity. (E) Sequence conservation. Sequence conservation is represented as mean PhastCons scores over all sites in the −/+1000 nt flanking region around the TSSs. PhastCons scores vary from 0 to 1, with 1 indicating high conservation. The X-axis shows the genomic position relative to the TSSs; the Y-axis shows the mean PhastCons scores. (F) Occurrence of repetitive elements in promoters divided by TSSD class. The X-axis shows the genomic position relative to the dominant peak of the TSSD; the Y-axis shows the number of nucleotides covered by respective repetitive elements, normalized by the number of TSSD. The transposable elements: LINE (top), LTR (middle) and SINE (bottom) are overrepresented in the ultra-dense core promoters (in blue) around the dominant TSS.

We started by investigating typical promoter-associated sequence patterns within or close to the TSSDs, including the INR pattern, the TATA-box and CpG islands using sequence logos, position weight matrices and UCSC genome browser annotation (see Methods) (Figure 3). Since we know that TATA-boxes are positively correlated by “tissue specific” promoters while CpG islands are associated with ubiquitously expressed genes, we also investigated the tissue specificity in the TSSDS by computing the relative entropy of the tags over the tissues [31] (Figure 3D) (see Methods). Consistent with the “broad” class of Carninci *et al.* [32], the scattered promoters have clear preference of a pyrimidine-purine (PyPu) initiator sequence at −/+1 sites (Figure 3A). These promoters are highly enriched for CpG islands, but despite this, a reasonable number of them have TATA-boxes, although the distribution of the TATA boxes are more spread than the “dense” class described below. Compared with the other classes, these promoters are the least tissue-specific as measured by relative entropy (Figure 3D; see Methods) and the most conserved in the promoter region, particularly −/+300 of the TSSs (Figure 3E; see Methods), as reported in [20]. Likewise, the “dense” class has many similarities with the “sharp” group suggested with Carninci *et al.* [32]: TATA-boxes are over-represented at the canonical location (−33 to −28 upstream of the TSS) in the dense group and CpG islands are less commonly overlapping these promoters. These promoters are more often tissue specific and tends to have higher evolutionary conservation upstream of the TSS compared to the other classes, consistent with previous studies [20]. Thus, the dense and scattered TSSD groups are roughly behaving as the SP and BR class of Carninci *et al.* [32]. The “ultra-dense” promoters do not have the pyrimidine at −1 but has a much stronger guanine at the +1 site. The guanine is very likely an artifact form the CAGE protocol, as described in [10]. These promoters are depleted of canonically placed TATA-boxes, and CpG islands are under-represented. This class is somewhere intermediate between the scattered and dense group in terms of tissue specificity (Figure 3D). Interestingly, these promoters are highly conserved downstream of the TSS but not upstream (Figure 3E and discussed further below).

#### Epigenetic patterns

**Figure 4:**
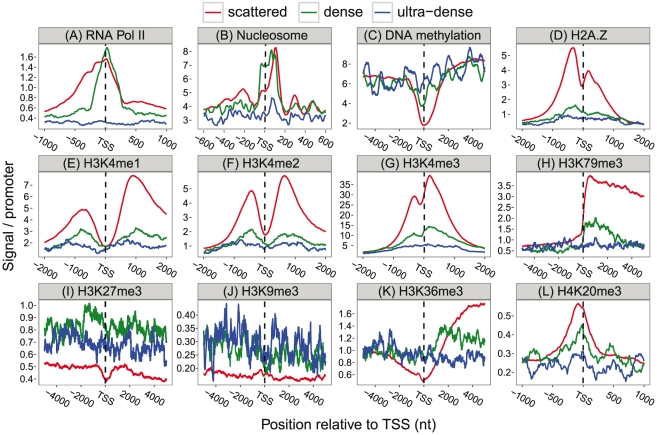
Epigenetic features of “scattered”, “dense” and “ultra-dense” TSSD subclasses. The genomic positions relative to the dominant TSS of each TSSD are labeled on the X-axis. The signal strength from respective epigenetic mark/feature is shown on the Y-axis, counted as ChIP tags/TSSD (or equivalent for non-ChIP approaches). The profiles are (A) RNA Polymerase II (B) Nucleosome positioning (center of nucleosome); (C) DNA methylation; (D) Histone variant H2A.Z; (E)–(L) Histone modifications. The RNA Pol II binding profile is from mouse ES cell while the epigenetic marks are from human CD4+T cell and mapped to mouse genome. See main text for discussion and Figure S5 for additional data.

We then assessed if the pattern of epigenetical marks around the TSSs of the three types of TSSDs are different by taking advantage of publically available ChIP-seq data sets, including DNA methylation from [33], histone modification and RNA polymerase II occupation from [34], [35], [36] and finally the overall nucleosomal positioning data from [37]. For all these sets, we examined the mean number of ChIP-ed tags for all nucleotides in the −/+5000 nt flanking region around the dominant peaks of the TSSDs and plotted the pattern at representative regions (Figure 4). An important caveat with this analysis is that while the CAGE data originates from many tissues, the ChIP data are from specific cells including human CD4+ T cells, mouse ES and NP cells for histone marks; human CD4+ T and mouse ES cells for RNA Pol II binding and human CD4+ for nucleosome occupancy. In cases where we use human ChIP data, we transferred CAGE tags from mouse to human using whole genome alignments (see Methods). While the cell sources differ, we see similar results for respective mark regardless of what cells that were used for the epigenetics experiments (Figure S4).

We find that RNA Pol II ChIP-seq data validates the difference in the widths of TSSDs, as the RNA Pol II distribution around the TSS of the “dense” promoters are more clearly defined and more condensed around the dominant peak (at the “TSS” position of the x-axis), than that of the “scattered” promoters (Figure 4A). RNA Pol II enrichment at the TSS and slightly downstream are clearly visible in both groups, although the dense group usually have slightly lower mean intensities, perhaps reflecting that many of these genes are tissue-restricted as noted above and therefore will be silent in the ChIP-ed cells. Interestingly, the RNA Pol II binding signal is almost suppressed in the ultra-dense promoters (discussed further below).

Likewise, we find that the DNA methylation around the dominant peak is strongly suppressed in scattered promoters, and slightly suppressed in dense promoters, while ultra-dense promoters show no distinct methylation patterns (Figure 4C). This agrees with the elevated signal of the CpG islands (Figure 3C) in the scattered promoters and the suppressed histone variant H2A.Z mark in dense/ultra-dense promoters (Figure 4D), since CpGs in promoters are commonly unmethylated (and therefore not repressed) [38] and H2A.Z signal is mutually exclusive to DNA methylation [39].

Similarly, we find the nucleosome positioning patterns (Figure 4B plots the inferred center position of nucleosomes from [37]) are strongly enriched in the scattered and dense promoters while depleted in the ultra-dense promoters. Interestingly, while both scattered and dense promoters show strong nucleosomal positioning for the nucleosomes positioned immediately downstream of the TSS (the so-called +1 nucleosome), the dense group has an additional peak immediately upstream of the TSS. This indicates that the TSS regions in many of these promoters are occupied by a nucleosome, and that the eviction of this nucleosome is likely an important part in their regulation. The scattered promoters have much less nucleosomal signal upstream of the TSS, and might therefore be dominated by open chromatin or at least have little nucleosomal positioning signals. These findings fit well with the tissue specificity findings above since the dense promoters need to be tightly controlled while the scattered promoters are often broadly expressed. This finding mirrors the suggestions made by Rach *et al.* [24], but the tendency here is stronger as the nucleosomal upstream peak was not shown by Rach *et al* since they focused on H2A.Z instead of generic nucleosomes.

We also find that the distributions of respective epigenetic marks around the dominant peaks of the scattered promoters are consistent with mRNA promoters as previously reported [34], [35], [36]. The scattered promoters (red in Figure 4) have elevated marks associated with transcription activation including H3K4me1/me2/me3 are highly enriched surrounding the TSS with a dip at the TSS (Figure 4E–G); this is also true for acetylation marks (Figure S5). The H3K79me3 mark, associated with active promoters in a narrow region surrounding TSS, has a very strong signal in the scattered promoters starting right at the TSS and continuing over the gene body (Figure 4H). The H3K36me3 mark, associated with elongation, is as expected strongly enriched at the transcribed region instead of at the TSS (Figure 4K). As these transcription activation-associated marks are strong in the scattered promoter class, it is logical that marks associated with transcriptional repression are depleted, e.g. H3K27me3 and H3K9me3 marks (Figure 4 I–J). The only exception to this is the H4K20me3 mark, a repressive mark in gene silencing mechanisms in mammals and associated with pericentric heterochromatin [40], has strong signal in the scattered promoters (Figure 4L). This may either be due to that some of the scattered promoters are silenced in the CD4 cells. Additional epigenetic mark distributions are shown in Figure S5.

The dense promoters have roughly the same epigenetic patterns as the scattered class, but a general trend is that the overall signal strength of respective marks is lower. This observation may be due to a lesser dependency of nucleosomal placement (as suggested in [24]), but may also be due to that the dense promoters to a much larger extent than the scattered promoters are expressed in restricted tissues, and therefore have few signals in the CD4 cells . As with TATA/CpG patterns, both typical activating and silencing promoter marks have little or no signal within the ultra-dense promoters (discussed further below).

### Sub-clustering of scattered TSSDs by number of peaks

As were surprised by the large diversity of distributions within the scattered class (which comprise about 90% of the dataset), we wondered if further subgroups exist within the data sets and what biological features that are responsible for these. We first investigated if these TSSds could be easily separated in sub-clusters based on simple distribution properties including peakedness, kurtosis and skew [41], without success(data not shown). Instead, we subdivided these TSSDs by how many clear peaks they have using a simple peak-calling algorithm (See Methods). We found that almost all (>99%) of these TSSDs could be classified as having 1 (∼34%), 2 (∼43%) ,3 (∼20%) or 4 (3%) peaks, and we focused on these in the below study. We then sought to investigate what biological features that were responsible for the number of peaks and their placement. The two most likely candidates are: i) the actual DNA sequence composition, as this in many cases can identify the most used TSS within a distribution [42]; ii) chromatin features, in particular nucleosome placement.

**Figure 5:**
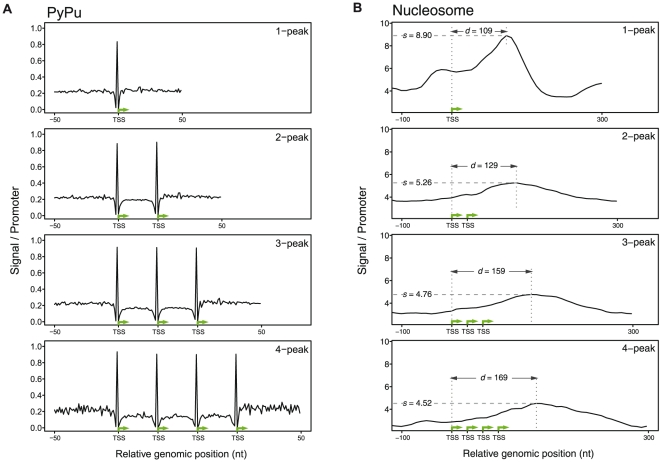
Sequence and expression features of subclasses within the scattered TSSDs. The plots show the scattered TSSDs (shown in Figure 2–4) divided by how many peaks they have. For each subclass, the TSSDs are aligned at their identified peak(s), denoted by green arrow(s), with the distance between two adjacent peaks rescaled to the same width in order to be comparable. The X-axis shows the genomic position relative to the peaks (TSS). The Y-axis shows the normalized signal per TSSD as in Figure 3. (A) Density of Pyrimidine-Purine (PyPu) dinucleotides, extended −50 nt at 5′ of the first peak and +50 nt 3′ of the last peak. Note that the PyPu dinucleotide enrichment is always positioned at −1/+1 nt of the peak(s), regardless of the number of peaks within the TSSD. (B) Density plot of nucleosome positioning, extended −100 nt at upstream of the first peak (the most 5′) and 300 nt downstream of the most 3′ peak. The nucleosome binding profile is from human CD4+T is plotted as in (Figure 4B). As in panel A), the distances between the TSSD peaks are rescaled to be the same in all TSSDs. In addition, *d* denotes the distance between the position of the highest nucleosome signal and the first peak. *s* denotes the scores of the binding intensity. Interestingly, the nucleosomal signal which is as expected at ∼+110 in the single peak TSSD is gradually shifted 20–30 nt downstream. In general, with more peaks the total nucleosomal positioning signals appears less distinct.

If the sequence content in the core promoter is the main underlying signal, we would expect a high over-representation of Pyrimidine-Purine (PyPu) dinucleotides at the +/−1 position, defined around respective peak(s), and possible also TATA-boxes, as these are the two motifs that have the greatest impact on TSS usage [42]. This is the case: there is a high PyPu signal pinpointing the peaks; regardless of the number of peaks: 80–90% of the peaks have this dinucleotide, compared to ∼22% for other positions (Fig. 5A). TATA-boxes are generally under-represented in the multi-peak TSSds but occurs more often in those scattered TSSds with only one peak(Figure S7). One interpretation of this is that the existence of a TATA box will make additional peak locations unfavorable.

At the same time the nucleosome occupation plots around the peaks show that the typical higher nucleosomal signal after a TSS (typically at around +110) is shifted so that it generally occurs after the last peak if multiple peaks exist (Figure 5B). The nucleosomal signal upstream of the peaks is generally very low, also for the most 5′ peak. This indicates that for scattered TSSDs with multiple peaks, most of the region is accessible for PIC formation, and the epigenetical signals are not indicative of the TSSD peak placements. Thus, the diversity observed in the scattered TSSDs is most likely explained by differences (and diversity) on sequence level between the different TSSDs in this group.

### Ultra-dense TSSDs are associated with ribosomal protein pseudogenes

The fact that the ultra-dense TSSDs lack both sequence and epigenetic features typically associated with promoters raised the question whether the ultra-dense TSSDs are caused by methodological noise, such as PCR bias and/or incorrect capture of cDNAs that are not full-length. One argument against random noise is that these TSSDs in most cases are composed of tags originating from more than one CAGE library (316 of the 323 ultra-dense TSSDs are composed of CAGE tags from 2 or more libraries). Another possible explanation is that these tags are mapping artifacts as we found that LINE, LTRS and SINE repeat elements are over-represented around the dominant TSS of TSSDs (Figure 3F) (see Methods).

If the DNA regions around ultra-dense TSSs are duplicated and identical, it would not be possible to map tags to them with the mapping protocol used, because only CAGE tags that map to a unique location are considered (we use the same mapping as in Carninci *et al.* [32] for consistency). Thus, one possibility is that a large part of the promoters are duplicated except for the ∼20 nt long window where the tag in the ultra-dense TSSD map uniquely, which would explain the sharp peak. We found that 20-mers in the region upstream of the TSS of ultra-dense regions are as mappable as 20-mers upstream of the two other classes, while mappability is decreased to ∼60% for tags starting one nucleotide downstream of the TSS (Figure S6). The reason for this is discussed further below.

We then investigated whether other experimental data supports the ultra-dense promoters, by overlapping them with publically available annotation data from GenBank [43], including 5′ EST and mRNA data (see Methods). While substantially fewer of the ultra-dense TSSDs are supported by RefSeq 5′ ends than dense and scattered TSSDs (8.67%, 54.79% and 88.26% of respective promoter set have at least one RefSeq 5′ end at the same strand within +/−300 nt of the dominant peak of the TSSD (Table S5)), 136 (46%) of the ultra-dense TSSDs that are not supported by mouse RefSeq annotations are supported by 5′ ends from mouse EST or mouse mRNA data, and 75 of them are also supported by 5′ ends from RefSeq genes of other species mapped to the mouse genome (the “Other RefSeq” track of the UCSC genome browser; see Methods and Table S5).

**Figure 6:**
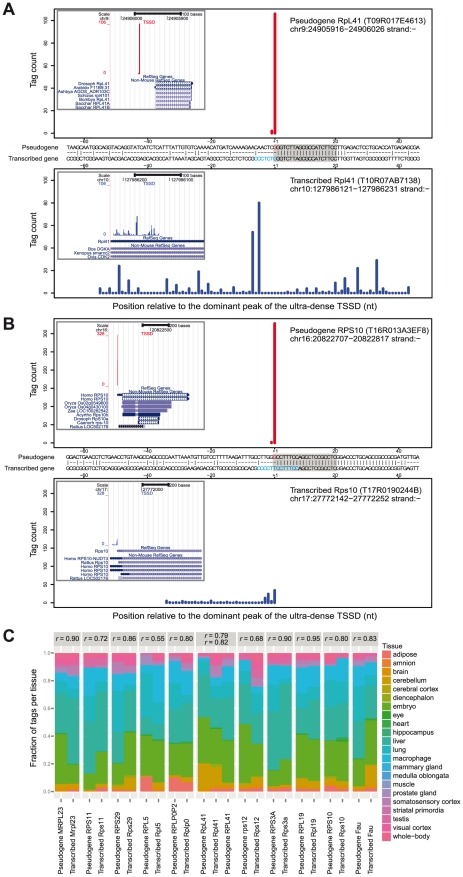
Ultra-dense TSSDs associated with ribosomal protein pseudo genes and their transcribed counterparts. (A)–(B) Examples of TSSDs mapping to processed pseudogenes and corresponding transcribed ribosomal protein gene promoters. Each example shows an alignment of the pseudogene (top) and transcribed gene (bottom) with the sequence alignment in the middle and a genome-browser view as the inset. In the browser view, the CAGE distribution (TSSD), Mouse RefSeq, RefSeq from other species are shown as separate tracks. Note that the pseudogene has an ultra-dense TSS distribution just at the inferred 5′ end of the pseudogene. In the alignment, the tag distributions (red for pseudogene; blue for transcribed gene) are aligned and shown with sequence comparison along the x-axis in the middle. The Y-axis shows the number of CAGE tags mapping at the region, only counting the 5′ end. Note that the regions upstream of the TSS are generally dissimilar while the +2∼+20 nt region from the TSS of the pseudogene TSSD is almost identical (covered by grey boxes). The CT-tract is colored in blue. The position of the single CAGE peak in the pseudogene coincides with the 1 nt difference just upstream of the CT track, where the pseudogene has a G (colored in red). (C) Correlation between pseudogene and transcribed gene CAGE tags in terms of distribution over tissues. The Y-axis shows the fraction of tags from each tissue as a stacked barplot for each TSSD. Each panel shows a pair of TSSD from the transcribed gene and the corresponding pseudogene. The transcribed Mouse Rpl41 gene has two corresponding pseudogenes with their own ultra-dense TSSD, and therefore has three columns instead of two. Spearman correlation coefficients comparing the tissue distributions of pseudogene and transcribed gene CAGE tags are shown above each panel. All of the correlations are statistically significant: P<0.01 in all cases (data not shown).

We noticed that 25 of the ultra-dense TSSDs that had no RefSeq support in mouse are overlapping ribosomal protein (RP) gene annotation from other species. In most of these cases, these genomic regions are processed pseudogenes, as the downstream gene has no introns and the indicated transcribed RP gene is in mouse mapping to another location – examples are shown in (Figure 6A).

Previous studies have identified 79 transcribed RP genes [44] and over 2000 RP pseudogenes [45] in the human genome. We noticed that the transcribed ribosomal genes in most cases had either a scattered or dense TSSD, while the pseudogene only had an ultra-dense TSSD. An important part of the explanation of this observation is that RP gene promoters have a substantially different promoter architecture compared to other genes in terms of sequence content with a CT-rich (oligopyrimidine tract) region around the TSS with transcription always started at a C residue (reviewed in [44]). Since the CT-rich region is a general feature of RP gene promoters, it will be harder to uniquely map CAGE tags to this region, which explains both the drop in mappability and higher evolutionary conservation in the immediate downstream region discussed above and shown in Figure S6 and Figure 3E.

There are two possible explanations for the ultra-dense TSSDs at the RP pseudogenes: either the pseudogene is transcribed as discussed in [46] or the ultra-dense TSS is erroneously mapped and in reality belongs to the genuine, transcribed RP gene.

While the first explanation cannot be ruled out completely using computational methods, it is unlikely since virtually all other promoters display a variance in their selection of TSSs – other ribosomal genes promoters generally have scatted or dense TSSDs. Moreover, the TSSDs lack epigenetic patterns reminiscent of expressed promoters (Figure 4)

We investigated the alternative explanation by aligning the core promoter region of pairs of a pseudogene and its corresponding transcribed RP gene. We found that in all 25 cases, there is one nucleotide edit distance between the corresponding region (+1∼+20 nt around the dominant peak of the ultra-dense TSSD promoters) in the pseudogene and the transcribed RP gene promoter. Of the cases , 24 have this 1-nt difference located the dominant peak of the pseudo promoter, in most cases located immediately upstream of the CT tract. In general, the pseudogene had a G instead of the original nucleotide in the transcribed gene (usually a C) (Figure 6A–B and Figure S8A–Y). The CAGE protocol has a known bias, where many CAGE tags have a nonspecific G at the most 5′ end, attributed to the template-free 5′-extension during the first-strand cDNA synthesis [47]. This would explain at least some of the ultra-dense TSSs found: pseudogenes that by chance has incorporated a G in or around the duplicated CT tract will have higher mapping scores than the originating region for tags having an added G, and this will result in a ultra-dense TSSD: tags mapping upstream of the transcribed RP gene will map to the correct, transcribed location since the upstream regions are dissimilar between gene and pseudogene while downstream tags might not be mapped at all, since the regions are almost identical. Thus, the ultra-dense TSSD is most probably originating from the dense or scattered distribution of the actual transcribed gene. Supporting this, the tissue distributions of CAGE tags in pseudogene and corresponding transcribed genes are highly correlated (Figure 6C and Table S6).

### Analysis on other data sets

To make sure our results are not specific to the dataset analyzed, we analyzed four additional sets: two tissue libraries from the FANTOM3 set in mouse from liver and embryo (2656 core promoters in liver set and 1341 core promoters in embryo set), the whole set of CAGE tags from human in FANTOM3 (5298 core promoters), and finally the FANTOM4 data set with 9201 core promoters from cell line THP-1 produced by a different sequencing technology (Table S1). Applying the same analysis as above, we in each of these sets identified the “scattered”, “dense” and “ultra-dense” TSSDs (Table S4). We did not observe any substantial differences between these sets, suggesting that the results are stable over tissues, species and sequencing techniques.

## Discussion

In this study we systematically investigated TSSDs to see how many stable groupings of such distributions that the data supports and compared these groups to previous classifications. Finally we examined what the biological properties of these groups were.

Our results indicate that in general, the three broad classes (BR, PB, MM) in [10] are in reality a gradient of distributions where it is hard to specify stable sub-classes. One way to interpret this diversity is that the actual TSS distribution has a limited direct role in defining the function of the downstream transcripts or is not actively regulated by for instance transcription factors, but rather is a function of local DNA sequence, since we know that the dinucleotides around individual TSSs to a large degree can predict the proportion of CAGE tags mapping there [19], and changes in dinucleotides between species can in many cases explain observed shifts in TSS distributions. This is further corroborated by the fact that if splitting up the scattered TSSDs in subgroups depending on how many peaks they have, simple dinucleotide counts can identify the location of each peak. In this light, the studies using two general “sharp” and “broad” classes are more motivated than more elaborate schemes.

However, we also find that the previously defined sharp class with certainty has two stable sub-classes with very different biological properties – one where there is a small spread of the TSSs around a dominant peak, perhaps reflecting the flexibility of the pre-initiation complex as discussed in [11], and one where essentially all CAGE tags map to the same nucleotide position. The first corresponds well to “text-book” core promoters dominated by TATA-boxes and tissue-specific genes, the other group lacks most biological signals associated with promoters – TATA-boxes, CpG islands and also typical epigenetic patterns such as H3K4me3 and RNA Polymerase II enrichment. Notably, such promoters have been included in a larger “sharp” category in previous studies. Thus, it is likely that these ultra-dense promoters are giving an undue influence on the “sharp “ class in such studies, as the depletion of promoter signals in the ultra-sharp sub-group will affect the average of the super-group.

There are two major explanation models for the ultra-sharp class: i) the TSSDs are due to either experimental of biological noise, such as mapping issues or recapping events or ii) these TSSDs represent atypical promoters.

We have shown that at least some of these TSSDs overlap with known ribosomal pseudogenes, and that this is likely due to mapping errors caused by the G-addition bias in the CAGE protocol: those TSSs likely belong to the genuine, transcribed RP gene. These problems are conceptually similar to cross-hybridization problems reported for hybridization-based methods, and have cautionary implications for mapping CAGE data – clearly, the G addition bias must be filtered at a stage before mapping [48], or a mapping strategy that considers the “stability” of a mapping should be considered: will the tag map to multiple locations if the first G is removed?

The same problem could potentially occur for any sequencing platform that uses short reads and where mismatches are tolerated in the mapping protocol. In particular, sequence methods that are trying to capture mRNA 5′ ends will be susceptible to this problem as reverse transcriptase often adds additional Gs once the end of the RNA is encountered – this feature is even used in certain protocols [49], [50], often refereed to as “template switching”.

Some of the newer mapping algorithms for small DNA reads use some of these concepts, for instance, Delve (Timo Lassmann, personal communication) and Sesam (as used in [51]) represent reads as weight matrices and can calculate the probability that a mapping is correct, weighting in all possible mappings and the difference between the strongest and the second strongest mapping.

Importantly, pseudo-RP gene promoters can only explain around 20% of these the ultra-dense TSSDs, and while other pseudogenes could cause similar issues, many of the ultra-dense TSSDs do not map close to obvious pseudogene candidates. Previous work have show the widespread use of repetitive elements working actual TSSs [52]; as we find an over-representation of repetitive elements in the ultra-dense promoters, this might account for some of the observations. Another explanation for these extremely sharp tag distributions is regulated cleavage and recapping, as suggested by Hoskins *et al.* [53]. Similarly, Mercer *et al.* [54] have suggested that many of intergenic CAGE tags are recapped partial mRNAs, based on the lack of epigenetic signals around such tags, and this and other studies ([10]) have shown specific expression patterns of (possibly) recapped transcripts within 3′ UTRs of genes. However, only a few of the ultra-dense TSSDs are overlapping internal exons (<5%), and none overlap the 3′ UTR, indicating that they represent another class of transcripts than those reported by Mercer *et al.* [54].

Regardless of their origin, our results show that it is highly relevant to separate these two peaked TSSDs from each other (or even filter out TSSDs composed of a single position) in any large-scale promoter analysis since they have very different properties and/or origin.

## Materials and Methods

### Data sources and data preprocessing

Our primary data set is the FANTOM3 data for 22 tissues of the May 2004 mouse (*Mus musculus*) draft genome data (mm5) obtained by cap analysis gene expression (CAGE) as defined [10]. Other data sets used in the analysis include FANTOM3 CAGE data of mouse liver (*Mus musculus*, mm5), FANTOM3 CAGE data of mouse embryo (*Mus musculus*, mm5), FANTOM3 CAGE data of human (*Homo sapiens Homo*, hg17), and FANTOM4 CAGE data of human (*Homo sapiens Homo*, hg18) [10], [55]. We applied the mappings of sequence tags from these studies and used their tag clusters (TCs) as our TSSDs, with the following additions: A TSSD is defined as a set of 5′ end of closely located tags that overlap each other at least 2 base pair (bp) on the same strand. The two-bp overlap is required in order to reduce the tiling effects observed in the original paper [10], where a requirement of one-bp overlap was used. In this study, we examined the TSSDs with no less than 100 tags. This threshold is set to yield a more accurate and robust clustering of TSSDs. However, a lower threshold is also acceptable if a data set has very few tags, as long as this threshold is the same throughout the study. We also applied Laplace's rule of succession to the TSSDs to reduce the background noise. The primary data set of all the tissues of *Mus musculus* contains 7,151,511 uniquely mapped tags and they yield 594,136 TSSDs as defined in previous study [10]. 7752 of the TSSDs with 5,463,328 tags fulfill our criterion and were used in further analysis. Similarly, we obtained 2656; 1341; 5298; 9201 TSSDs from other data set as described in Table S1.

### GM-Distance: Measuring dissimilarity between distributions

We represented a core promoter in the format of a TSSD, which displays a histogram of the occurrence of the 5′ end of CAGE tags at each genomic position. In order to define groups of similar TSS distributions, we needed to define a sensible way to measure the similarity, or distance, between TSSDs. We based our metric on: the minimum difference of pair assignments (MDPA) [56], which is a true metric. MDPA is a distance between sets of equal size. Given two ordinal type histograms [56] of *n* elements in *b* bins, A and B, the MDPA between them can be calculated as the necessary cell movements to transform one histogram into the target histogram. For instance, a histogram A can be transformed into B by moving elements to left or right and the total of all necessary minimum movements is the distance between them. In our study, the TSSDs are ordinal histograms, representing the genomic positions of the tags. However, the MDPA method requires that the numbers of bins in the two histograms are identical, and the total numbers of objects falling into all bins are the same. This is not true for most pairwise comparisons of TSSDs. To fulfill the requirement we normalized the distributions to sum up to one for each TSSD, which also makes them comparable in shape instead of in magnitude as noted above. To account for variable lengths of TSSDs, we let one distribution slide over the other and padded distributions with tag counts of 0 when needed (i.e. equal number of bins). In addition, we also took strand information into account, so that all distributions are in the sense direction (i.e. from 5′ to 3′) before comparing them.

### Unsupervised clustering

Hierarchical clustering was computed with the R language function *hclust* with Ward linkage. The method was fed a 7752-by-7752 dissimilarity matrix of all TSSDs (using GM-distance as described above) as input. A produced tree object was then cut by R function cutree with a specified number of clusters (*k*) to split the tree into *k* clusters. *k*-medoids and *k*-means clustering is described in Text S1, but was also made with corresponding standard R methods.

### Stability of a clustering and the resulting clusters

We employed the mean Jaccard coefficient to measure the stability of each cluster from the clustering by bootstrap resampling in [30]. By resampling the original data with replacement B = 100 times we obtained B pseudo data sets. For each pseudo data set 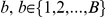, we obtained a pseudo clustering using the same procedure as for the original clustering based on the original data set. For each original cluster 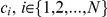 (where 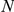 is the total number of clusters in the original clustering), its stability was represented by mean Jaccard coefficient as
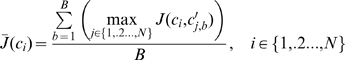
(1)where 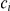 is a cluster of TSSDs in the original clustering; 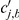 is the most similar cluster of 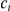 in a pseudo clustering b; B is the number of bootstrap samples; 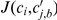 is the Jaccard (similarity) coefficient of 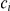 and 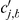, which is defined as the ratio of the size of their set intersection and that of their set union,
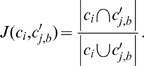
(2)


For each original cluster 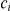, we took the average of the Jaccard coefficients 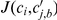 over the resampled data sets *b* as a measurement of its stability.

We collected summary statistics for the stability of the overall clusters to infer the overall robustness among different clustering scenarios. We then proposed a stability measure of one clustering 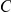 and computed it by the weighted mean of the cluster mean Jaccard coefficients 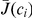 across all cluster 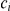, with the coefficient of each cluster weighted by the cluster size 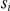 (i.e. the number of the TSSDs in the cluster). This stability measure of a clustering is denoted by 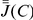 and formulated as
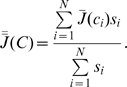
(3)


### Peakedness of TSSD distributions

#### Individual peakedness score

The peakedness of a distribution (TSSD) *g* was defined as
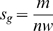
(4), where *m* is the tag count at the dominant peak (the mode); *n* is the total number of tags in the distribution; *w* is the width of the distribution, i.e. the number of nucleotides covered. This statistic measures similar features as kurtosis [41].

#### Intra-cluster peakedness score

The intra-cluster peakedness score was calculated by taking the average across all peakedness scores of every TSSDs in a cluster.

### Two-level hybrid clustering to find stable clusters

We proposed a two-level clustering approach, attempting to find homogeneous clusters from a highly heterogeneous dataset. The approach is composed of a hierarchical clustering at the first level and a k means partition at the second. We first constructed a 7752-by-7752 dissimilarity matrix on the TSSDs in the primary data set and then push the dissimilarity matrix into a hierarchical clustering procedure (as described above). We obtained 500 clusters by partitioning the dendrogram with a cutline (*k_1_* = 500). Next, we calculated 500 intra-cluster peakedness scores and applied a *k*-means clustering procedure (using R function kmeans) to aggregate them into three larger groups by fitting an optimal model with *k_2_* = 3 (see Results section).

### Peak identification in scattered TSSDs

Our peak identification algorithm is controlled by two parameters: span and intensity. Span is the span (the width) of a single peak. We set span to 20 nt, which also means peaks within half of the value (10 nt) will be counted as one peak. Intensity is defined as the relative peak intensity against the total number of tags. We set the intensity threshold to 0.05. That is, a peak is identified when the tag count at a specific nucleotide position has no less than 5% of total tags in a TSSD. If no peak is detected, we classified the TSSD as “uniform”. This occurred for less than 1% of the scattered TSSDs.

### Characterization of TSSDs using biological meta-data

#### TATA patterns

The promoters and the flanking region (+/−100 nt) around their dominant peaks were scanned with the TATA position weight matrix (PWM) from JASPAR database (ID: POL012.1; [57]). The TATA patterns were then determined given scores above a threshold of 70%, as described in [58]. For each prediction, we tabulated the position of the 1^st^ T in the TATA box and calculated the density per unique TSSD in each group.

#### CpG Islands

The promoters were aligned at the dominant peaks. For each TSSD, the position of the CpG islands to the dominant peaks was examined in the flanking regions (+/−300 nt). Then we count the number of the CpG islands covered at each position for each groups of TSSDs and normalized the number by the total number of TSSDs in that group (in normalized units of sites per TSSD). The annotation of CpG islands were retrieved from UCSC genome browser track generated according to [59].

#### Tissue specificity

The overall and categorical tissue specificity were calculated based on relative entropy, also known as Kullback-Leibler divergence as in [11]:
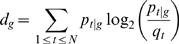
(5)where *t* denotes a tissue (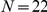), 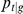 is the distribution the tissues in the tags of a TSSD 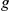; 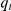 is the distribution the tissues in all tags. This minimum distance d is 0 when 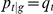; i.e. when the distribution of the tissues in one TSSD resembles the distribution in the whole data, the distance is low, which indicates low tissue specificity.

#### Sequence conservation

Sequence conservation was calculated as mean PhastCons [60], [61] scores over all sites with the promoters aligned at the dominant peaks. For each TSSD, the conservation intensity was examined in the flanking regions (+/−1000 nt) of their TSSs. Then we sum up the PhastCons scores at each position for each group of TSSDs and normalized the value by the total number of TSSDs in that group. The PhastCons scores were retrieved from UCSC genome browser [62], which is determined by alignments of 4 vertebrate genomes (Rat, Human, Dog and Chicken) with Mouse.

#### Genomic annotation

The original CAGE data was taken from Carninci et al. [32], and thus mapped to the mm5 assembly (the May 2004 Mus musculus draft genome data). We did not remap this set to a newer assembly to be able to compare with the original results present in Carninci et al. [32]. However, the additional CAGE data sets analyzed were mapped to newer assemblies and show the same general findings). As some data tracks are only present in newer assemblies (such as the “Other RefSeq” track), we used the LiftOver [62] tools to transfer annotation from mm9 to mm5 using standard settings.

#### Epigenetic patterns

Epigenetic marks were obtained from publically available ChIP-seq data sets, including DNA methylation [33], histone modification and RNA polymerase II occupation [34], [35], [36], [63] and the nucleosomal positioning [37].

The DNA methylation was obtained from the Sequence Read Archive (SRA; [64]), pooled by three replicate samples of human embryonic stem cells (SRA accessions SRX020007, SRX020008, SRX020009; Chavez, Jozefczuk *et al.* 2010). The SRA submissions were then converted to FASTQ files by SRA toolkit [64] and mapped to human genome (hg18) by bowtie [65]. Finally, the immuno-enriched areas were identified using MACS [66] based on uniquely mapped reads and then transferred to mouse genome (mm5) using LiftOver [62]. The enriched regions of histone lysine and arginine methylations as well as histone variant H2A.Z and RNA polymerase II in CD4+ T cell were provided by [34], [35], [36]. The alignment coordinates of the sequence reads for histone modifications in mouse ES and NP cells (mm8) were obtained from [35], [63] and then the immuno-enriched areas were identified by MACS [66] based on uniquely mapped reads and then transferred to mouse genome (mm5). The read coverage of the nucleosome was calculated according to average nucleosome dyad positions [67] without smoothing. For all these sets, we counted the number of ChIP-ed tags for all nucleotides in the flanking region (−/+5000 nt or −/+1000 nt) around the dominant peak of the TSSDs. Aligning the TSSDs at the dominant peaks, we computed densities as a ratio of reads per unique TSSD for all groups, in normalized units of “reads per TSSD (promoter)”.

## Supporting Information

Text S1 **Unsupervised clustering using k-medoids.**
(PDF)

Table S1 **CAGE data sets analyzed in the study, including mouse whole-body, liver, embryo and human whole-body libraries from FANTOM3; and human THP1 libraries from FANTOM4.**
(PDF)

Table S2 **The clustering stability measure of five data sets by (A) hierarchical clustering and (B)** ***k***-**medoids.**
(PDF)

Table S3 **Cluster analysis of five data sets by two-level clustering.**
(PDF)

Table S4 **CAGE tags mapped to different genomic regions.**
(PDF)

Table S5 **RefSeq annotation of TSSDs. EST/mRNA support of the transcribed and pseudo- RP-gene promoter TSSDs.** EST, mRNA and “Other RefSeq” evidence for mouse RefSeq-unannotated TSSDs.
(PDF)

Table S6 **Correlation of the tissue distributions between the pseudogene TSSDs and the corresponding transcribed-gene TSSDs.** *r* denotes the Spearman correlation coefficient.
(PDF)

Figure S1 **Explained variance by 1^st^-level clustering.** Explained variance (Y-axis) of five data sets modeled by (A) hierarchical clustering and (B) *k*-medoids clustering given number of clusters (*k*, X-axis).
(PDF)

Figure S2 **Explained variance of the 2^nd^-level clustering, modeled by** ***k*** **-means.**
(PDF)

Figure S3 **Tiling effect at borders of TSSDs.** Examples of TSSDs that are labeled “scattered” by our method but SP (“sharp peak”) in Carninci *et al.*, due to tiling effects of tags spreading around the dominant peak.
(PDF)

Figure S4 **Similar patterns of epigenetic marks between cell lines.** Comparison of epigenetic patterns across between different cell lines, for the different promoter classes. Regardless of what cell that is used as reference, the distributions are similar. (A)–(D) human CD4+ T cell. (E)–(H) mouse ES cell. (I)–(K) mouse NP cell.
(PDF)

Figure S5 **Additional epigenetic mark densities (generated in a similar procedure as in** **Figure 4** **).**
(PDF)

Figure S6 **Sequence mapping uniqueness.** Genomic sequence mapping uniqueness around the TSSs, by sampling and mapping 20-mer DNA fragments around the TSSs.
(PDF)

Figure S7 **TATA motifs of subclasses of “scattered” TSSDs.** Density plot of TATA-box extend −50 nt at 5′ of the first peak and 20 nt at 5′ of the last peak. The X-axis shows the genomic position relative to the peaks (TSS, indicated by a green arrow). The Y-axis shows the number of predicted sites per TSSD, as in Figure 3B. TATA motifs are dominant at around −30 nt in the 1-peak “scattered” TSSDs (top panel) while are strongly weakened in other subclasses. For each subclass, the TSSDs are aligned at their identified peaks, with the distance between two adjacent peaks rescaled to same width.
(PDF)

Figure S8 **Alignments of pseudo and transcribed genes promoters.** (A)–(Y) Alignments between pseudogene promoters (top) and the corresponding transcribed ribosomal protein gene promoters (bottom), as in Figure 5. Their sequences are aligned along the X-axis in between. X-axis shows the tag 5′ end position relative to the dominant peak (at position 0) of the pseudogene TSSDs; Y-axis shows the count of 5′ ends of the tags.
(PDF)
