# Supplementary material for: Systematic Clustering of Transcription Start Site Landscapes
Source: PLoS One. 2011 Aug 24;6(8):e23409. doi: 10.1371/journal.pone.0023409 (PMC3160847; doi:10.1371/journal.pone.0023409)
Supplement: Text S1 — Unsupervised clustering using k-medoids. (PDF) [file pone.0023409.s001.pdf]

Text S1. Unsupervised clustering using *k*-medoids

In the 1st-level clustering, we also applied the *k*-medoids technique (using R function *pam* in the “cluster” package) to make certain that the clustering results are not specific to the clustering method. The difference to the hierarchical method employed above is that the method requires the number of groups (*k*) defined in advance. We then examined how the clustering model of *k*-medoids fits the data by the “elbow” criterion, which is consistent with the general finding in the hierarchical clustering and we observe the same heterogeneity using *k*-mediods. Supplementary Figure S1B shows the variance explained by the model.
