# Supplementary material for: Systematic Clustering of Transcription Start Site Landscapes
Source: PLoS One. 2011 Aug 24;6(8):e23409. doi: 10.1371/journal.pone.0023409 (PMC3160847; doi:10.1371/journal.pone.0023409)
Supplement: Table S1 — CAGE data sets analyzed in the study, including mouse whole-body, liver, embryo and human whole-body libraries from FANTOM3; and human THP1 libraries from FANTOM4. (PDF) [file pone.0023409.s002.pdf]

Table S1. CAGE data sets

Data set for clustering	Number of TSSDs (with no less than 100 tags)	Number of tags
FANTOM3 Mouse	7752	5463328
FANTOM3 Mouse liver	2656	1491547
FANTOM3 Mouse embryo	1341	643883
FANTOM3 Human	5298	3664127
FANTOM4 Human	9201	11030288 (TPM)
