# Supplementary material for: Systematic Clustering of Transcription Start Site Landscapes
Source: PLoS One. 2011 Aug 24;6(8):e23409. doi: 10.1371/journal.pone.0023409 (PMC3160847; doi:10.1371/journal.pone.0023409)
Supplement: Table S2 — The clustering stability measure of five data sets by (A) hierarchical clustering and (B) k-medoids. (PDF) [file pone.0023409.s003.pdf]

Table S2. Clustering stabilities

A. Hierarchical clustering

	Hierarchical clustering				
k	**FANTOM3 Mouse**	**FANTOM3 Human**	**FANTOM3 Mouse embryo**	**FANTOM3 Mouse liver**	**FANTOM4 Human**
2	0.73	0.78	0.74	0.78	0.91
3	0.61	0.65	0.74	0.67	0.66
4	0.63	0.54	0.71	0.57	0.65
5	0.58	0.59	0.63	0.55	0.56
6	0.54	0.53	0.61	0.51	0.55
7	0.52	0.52	0.58	0.48	0.53
8	0.48	0.50	0.51	0.48	0.53
9	0.47	0.48	0.50	0.48	0.55
10	0.46	0.47	0.50	0.47	0.55
15	0.43	0.46	0.48	0.46	0.50
20	0.41	0.40	0.48	0.45	0.44
25	0.40	0.42	0.49	0.42	0.43
30	0.38	0.42	0.49	0.42	0.42
40	0.37	0.39	0.49	0.42	0.42
50	0.34	0.39	0.47	0.42	0.41

B. *k*-medoids

	***k*-medoids**				
**k**	**FANTOM3 Mouse**	**FANTOM3 Human**	**FANTOM3 Mouse embryo**	**FANTOM3 Mouse liver**	**FANTOM4 Human**
2	0.95	0.99	0.97	0.99	0.99
3	0.93	0.91	0.93	0.95	0.97
4	0.85	0.89	0.86	0.83	0.92
5	0.85	0.80	0.80	0.79	0.86
6	0.73	0.75	0.73	0.67	0.84
7	0.80	0.80	0.64	0.67	0.80
8	0.66	0.64	0.65	0.63	0.80
9	0.61	0.69	0.64	0.62	0.67
10	0.58	0.63	0.63	0.59	0.71
15	0.57	0.58	0.57	0.51	0.74
20	0.55	0.53	0.55	0.49	0.62
25	0.54	0.54	0.57	0.47	0.58
30	0.52	0.53	0.57	0.52	0.55
40	0.49	0.52	0.50	0.51	0.52
50	0.48	0.46	0.49	0.49	0.53
