# Supplementary material for: Systematic Clustering of Transcription Start Site Landscapes
Source: PLoS One. 2011 Aug 24;6(8):e23409. doi: 10.1371/journal.pone.0023409 (PMC3160847; doi:10.1371/journal.pone.0023409)
Supplement: Table S3 — Cluster analysis of five data sets by two-level clustering. (PDF) [file pone.0023409.s004.pdf]

Table S3. Cluster analysis of five data sets by two-level clustering

Data set for clustering	TSSDs			Total TSSDs
	Scattered	Dense	Ultra-dense	
FANTOM3 Mouse	7095 (91.5%)	334 (4.3%)	323 (4.2%)	7752
FANTOM3 Mouse liver	2398 (92.3%)	149 (5.6%)	109 (4.1%)	2656
FANTOM3 Mouse embryo	1097 (81.8%)	152 (11.3%)	92 (6.7%)	1341
FANTOM3 Human	4749 (89.6%)	247 (4.7%)	302 (5.7%)	5298
FANTOM4 Human	7987 (86.8%)	516 (5.6%)	698 (7.6%)	9201
