# Supplementary material for: Systematic Clustering of Transcription Start Site Landscapes
Source: PLoS One. 2011 Aug 24;6(8):e23409. doi: 10.1371/journal.pone.0023409 (PMC3160847; doi:10.1371/journal.pone.0023409)
Supplement: Table S4 — CAGE tags mapped to different genomic regions. (PDF) [file pone.0023409.s005.pdf]

Table S4. CAGE tags mapped to different genomic regions

Group	TSS(-300~+301nt)	Upstream(-2000~-299nt)	3'-UTR	Exon	Intron
scattered	5951	234	95	4088	378
dense	175	13	20	132	26
ultra-dense	22	3	3	17	43
