# Supplementary material for: Systematic Clustering of Transcription Start Site Landscapes
Source: PLoS One. 2011 Aug 24;6(8):e23409. doi: 10.1371/journal.pone.0023409 (PMC3160847; doi:10.1371/journal.pone.0023409)
Supplement: Table S5 — RefSeq annotation of TSSDs. EST/mRNA support of the transcribed and pseudo- RP-gene promoter TSSDs. EST, mRNA and “Other RefSeq” evidence for mouse RefSeq-unannotated TSSDs. (PDF) [file pone.0023409.s006.pdf]

Table S5. TSSD annotation

A. RefSeq annotation of TSSDs

group	RefSeq-annotated TSSDs (%)	RefSeq-unannotated TSSDs (%)	Total
scattered	6262 (88.26%)	833 (11.74%)	7095
dense	183 (54.79%)	151 (45.21%)	334
ultra-dense	28 (8.67%)	295 (91.33%)	323

B. EST/mRNA support of the transcribed and pseudo- RP-gene promoter TSSDs

group	Count of RefSeq-annotated RP-gene promoters, the count of these promoters with mouse EST/mRNA support, their ratio in percentage	Count of RP pseudogene promoters (annotated by "Other RefSeq"), the count of these promoters with mouse EST/mRNA support, their ratio in percentage
scattered	122, 110, 90.16%	833, 4, 0.48%
dense	3, 2, 66.67%	151, 5, 3.31%
ultra-dense	2, 2, 100%	295, 25, 8.47%

C. EST, mRNA and "Other RefSeq" evidence for mouse RefSeq-unannotated TSSDs.

group	TSSDs without mouse RefSeq annotation			
	Total	TSSDs associated with EST/mRNA (%)	TSSDs associated with EST/mRNA and gene evidence from other species (%)	TSSDs associated with EST/mRNA and RP-gene evidence from other species (%)
scattered	833	805 (96.64%)	371 (44.54%)	4 (0.48%)
dense	151	69 (45.7%)	17 (11.26%)	5 (3.31%)
ultra-dense	295	136 (46.1%)	75 (25.42%)	25 (8.47%)
