# Supplementary material for: Systematic Clustering of Transcription Start Site Landscapes
Source: PLoS One. 2011 Aug 24;6(8):e23409. doi: 10.1371/journal.pone.0023409 (PMC3160847; doi:10.1371/journal.pone.0023409)
Supplement: Table S6 — Correlation of the tissue distributions between the pseudogene TSSDs and the corresponding transcribed-gene TSSDs. r denotes the Spearman correlation coefficient. (PDF) [file pone.0023409.s007.pdf]

Table S6. Tissue usage correlations

Pseudogene	Transcribed gene	*r*	P-value
Pseudogene MRPL23	Transcribed Mrpl23	0.900	1.246E-08
Pseudogene RPS11	Transcribed Rps11	0.721	1.519E-04
Pseudogene RPS29	Transcribed Rps29	0.861	2.650E-07
Pseudogene RPL5	Transcribed Rpl5	0.546	8.591E-03
Pseudogene RPLP0P2	Transcribed Rplp0	0.803	6.831E-06
Pseudogene RpL41	Transcribed Rpl41	0.794	1.041E-05
Pseudogene RPL41	Transcribed Rpl41	0.824	2.417E-06
Pseudogene rps12	Transcribed Rps12	0.683	4.635E-04
Pseudogene RPS3A	Transcribed Rps3a	0.898	1.439E-08
Pseudogene RPL19	Transcribed Rpl19	0.949	1.634E-11
Pseudogene RPS10	Transcribed Rps10	0.799	8.107E-06
Pseudogene Fau	Transcribed Fau	0.830	1.727E-06
