# Supplementary material for: Systematic Clustering of Transcription Start Site Landscapes
Source: PLoS One. 2011 Aug 24;6(8):e23409. doi: 10.1371/journal.pone.0023409 (PMC3160847; doi:10.1371/journal.pone.0023409)

Figure S1. Explained variance by 1st-level clustering

A. Hierarcical clustering

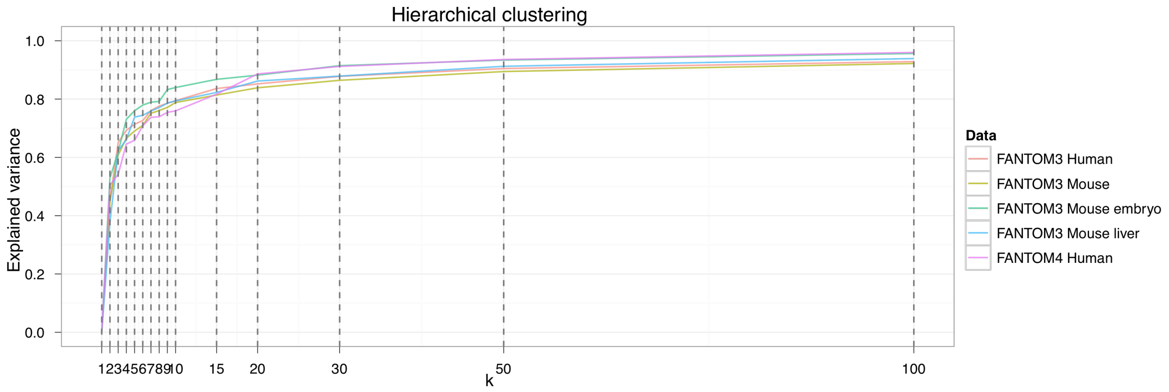

B. *k*-medoids clustering

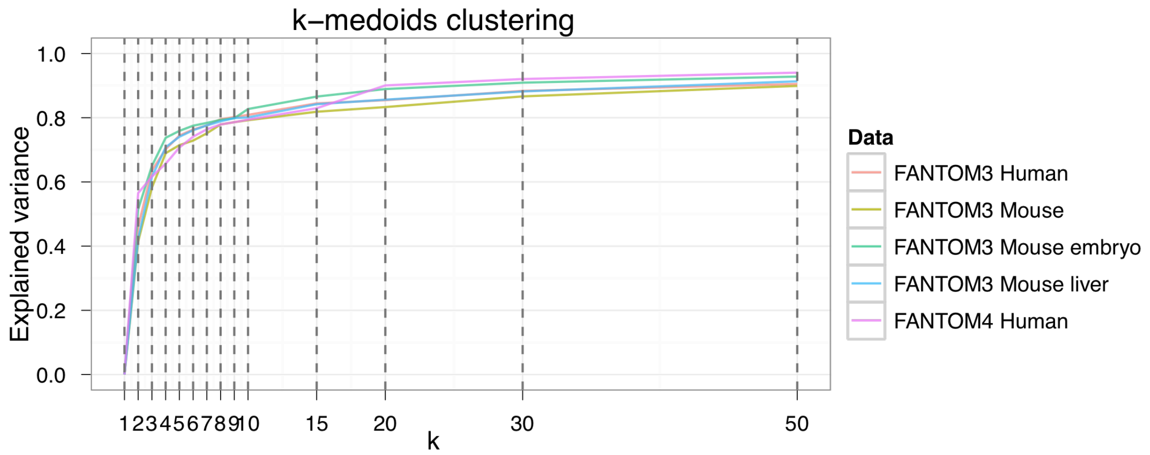

Supplement: Figure S1 — Explained variance by 1st-level clustering. Explained variance (Y-axis) of five data sets modeled by (A) hierarchical clustering and (B) k-medoids clustering given number of clusters (k, X-axis). (PDF) [file pone.0023409.s008.pdf]
