# Supplementary figures and images for: Systematic Clustering of Transcription Start Site Landscapes
Source: PLoS One. 2011 Aug 24;6(8):e23409. doi: 10.1371/journal.pone.0023409 (PMC3160847; doi:10.1371/journal.pone.0023409)

**Figure S2. Explained variance by 2<sup>nd</sup>-level clustering (*k*-means)**

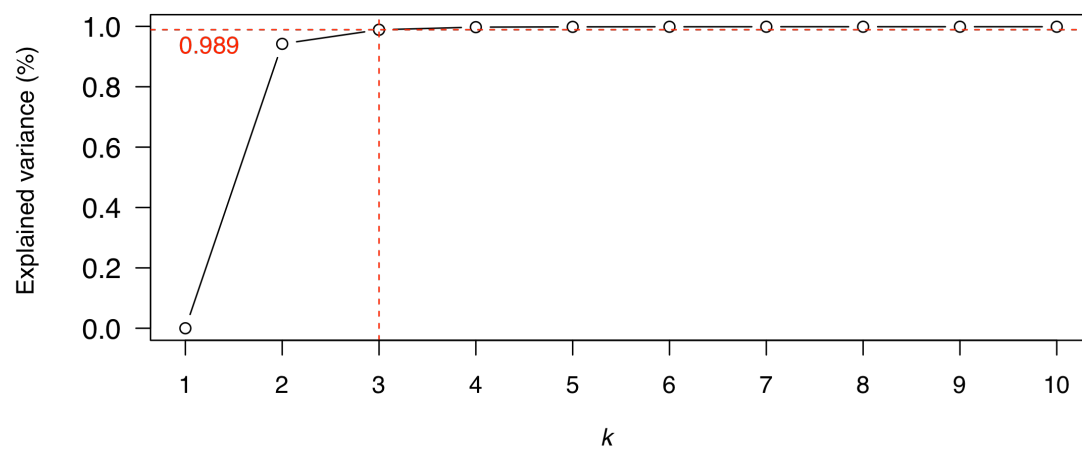

Supplement: Figure S2 — Explained variance of the 2nd-level clustering, modeled by k -means. (PDF) [file pone.0023409.s009.pdf]

**Figure S3. Tiling effect at borders of TSSDs**

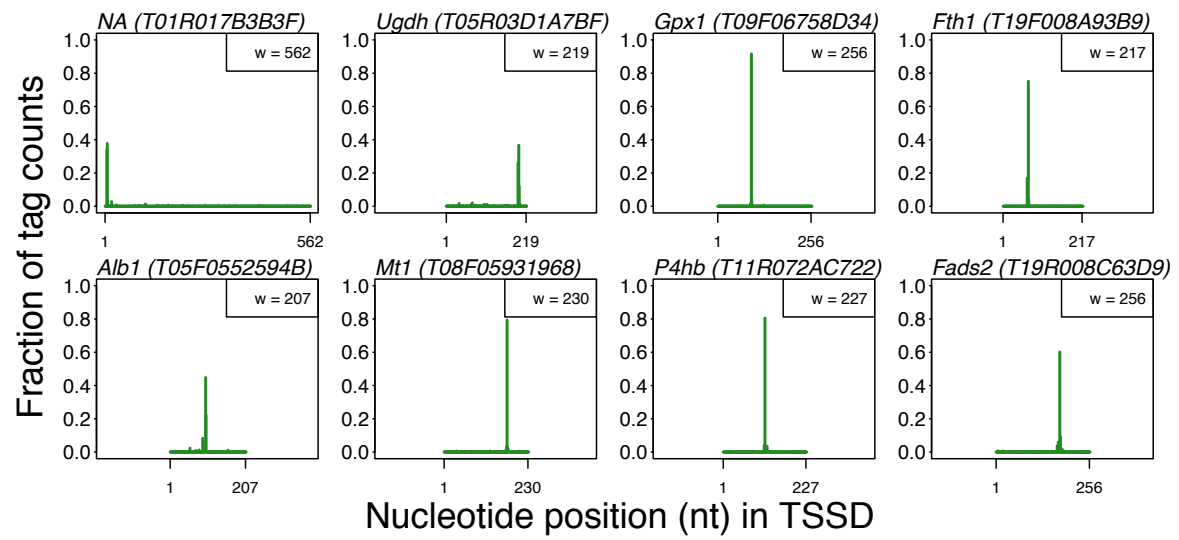

Supplement: Figure S3 — Tiling effect at borders of TSSDs. Examples of TSSDs that are labeled “scattered” by our method but SP (“sharp peak”) in Carninci et al., due to tiling effects of tags spreading around the dominant peak. (PDF) [file pone.0023409.s010.pdf]

**Figure S4. Similar patterns of epigenetic marks between cell lines**

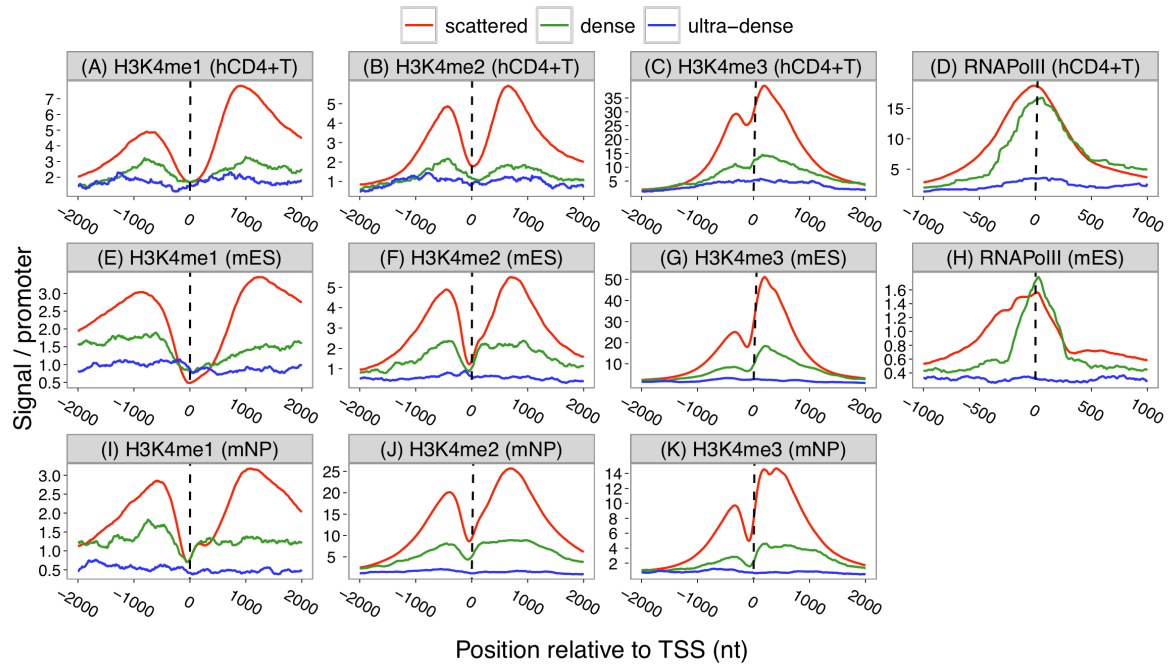

Supplement: Figure S4 — Similar patterns of epigenetic marks between cell lines. Comparison of epigenetic patterns across between different cell lines, for the different promoter classes. Regardless of what cell that is used as reference, the distributions are similar. (A)–(D) human CD4+ T cell. (E)–(H) mouse ES cell. (I)–(K) mouse NP cell. (PDF) [file pone.0023409.s011.pdf]

**Figure S5. Patterns of epigenetic marks**

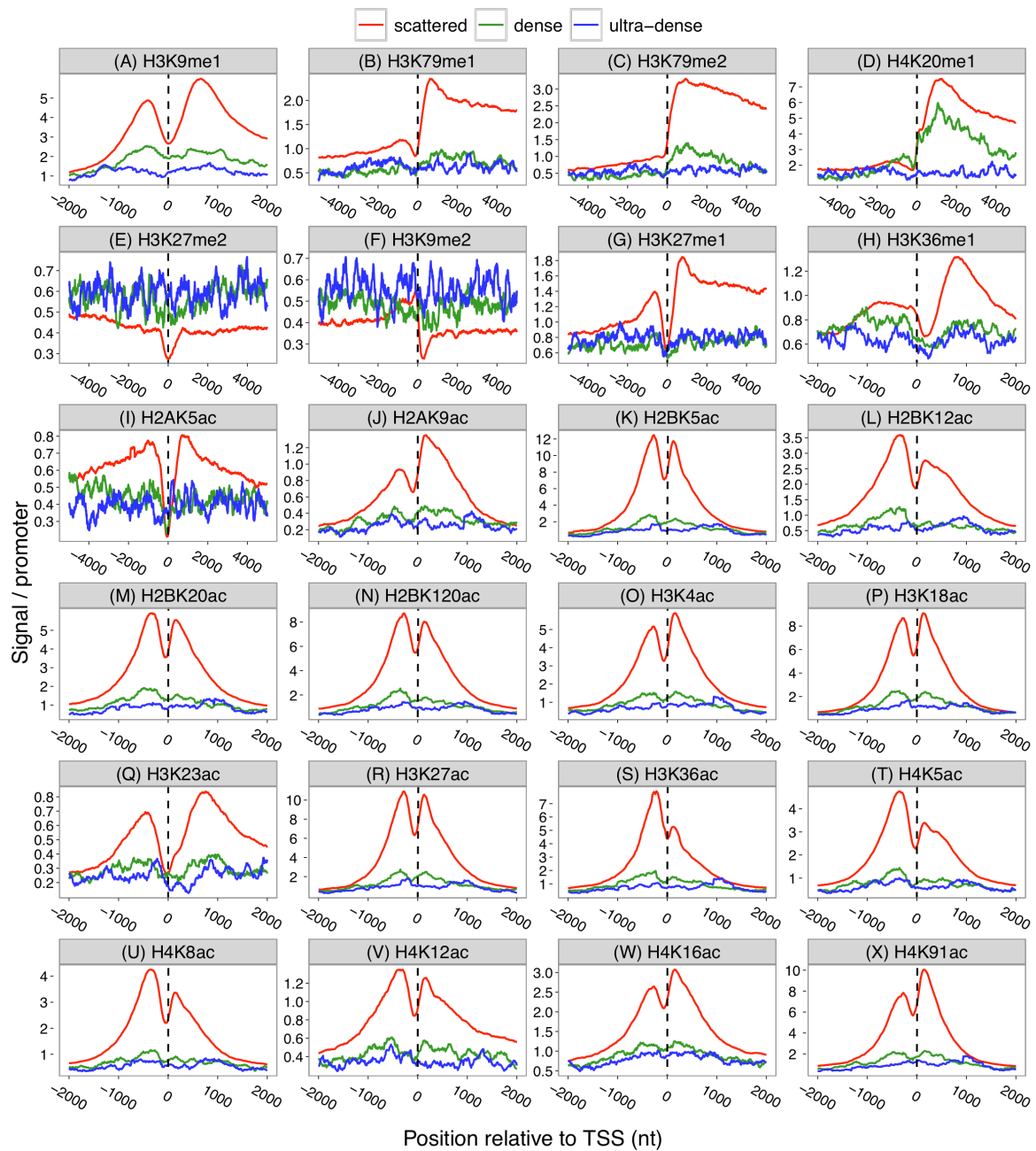

Supplement: Figure S5 — Additional epigenetic mark densities (generated in a similar procedure as in Figure 4 ). (PDF) [file pone.0023409.s012.pdf]

**Figure S6. Sequence mapping uniqueness**

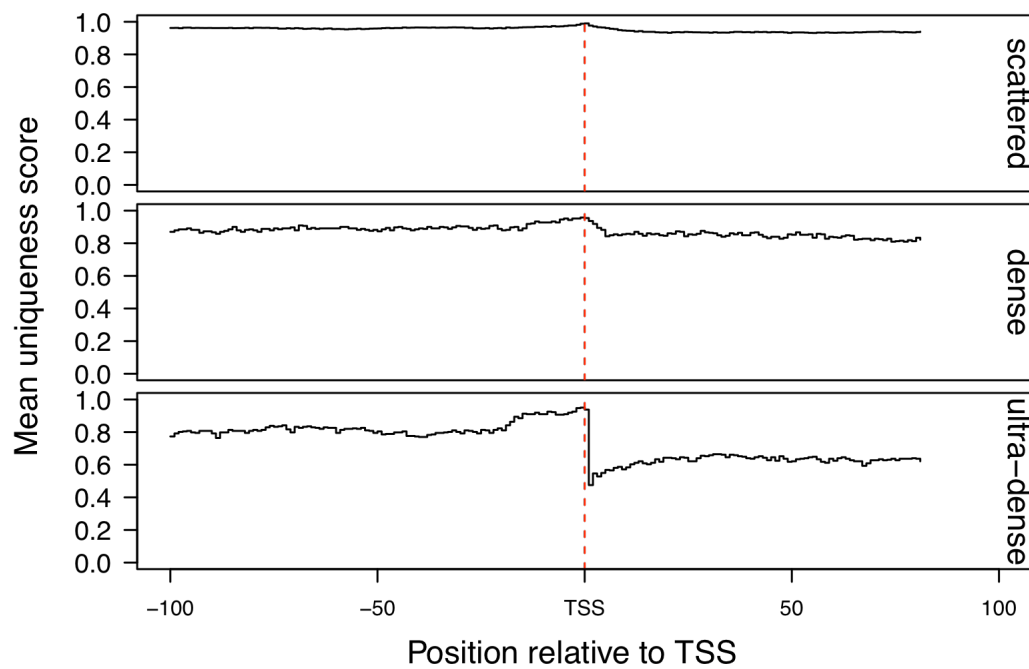

Supplement: Figure S6 — Sequence mapping uniqueness. Genomic sequence mapping uniqueness around the TSSs, by sampling and mapping 20-mer DNA fragments around the TSSs. (PDF) [file pone.0023409.s013.pdf]

**Figure S7. TATA motifs of subclasses of “scattered” TSSDs.**

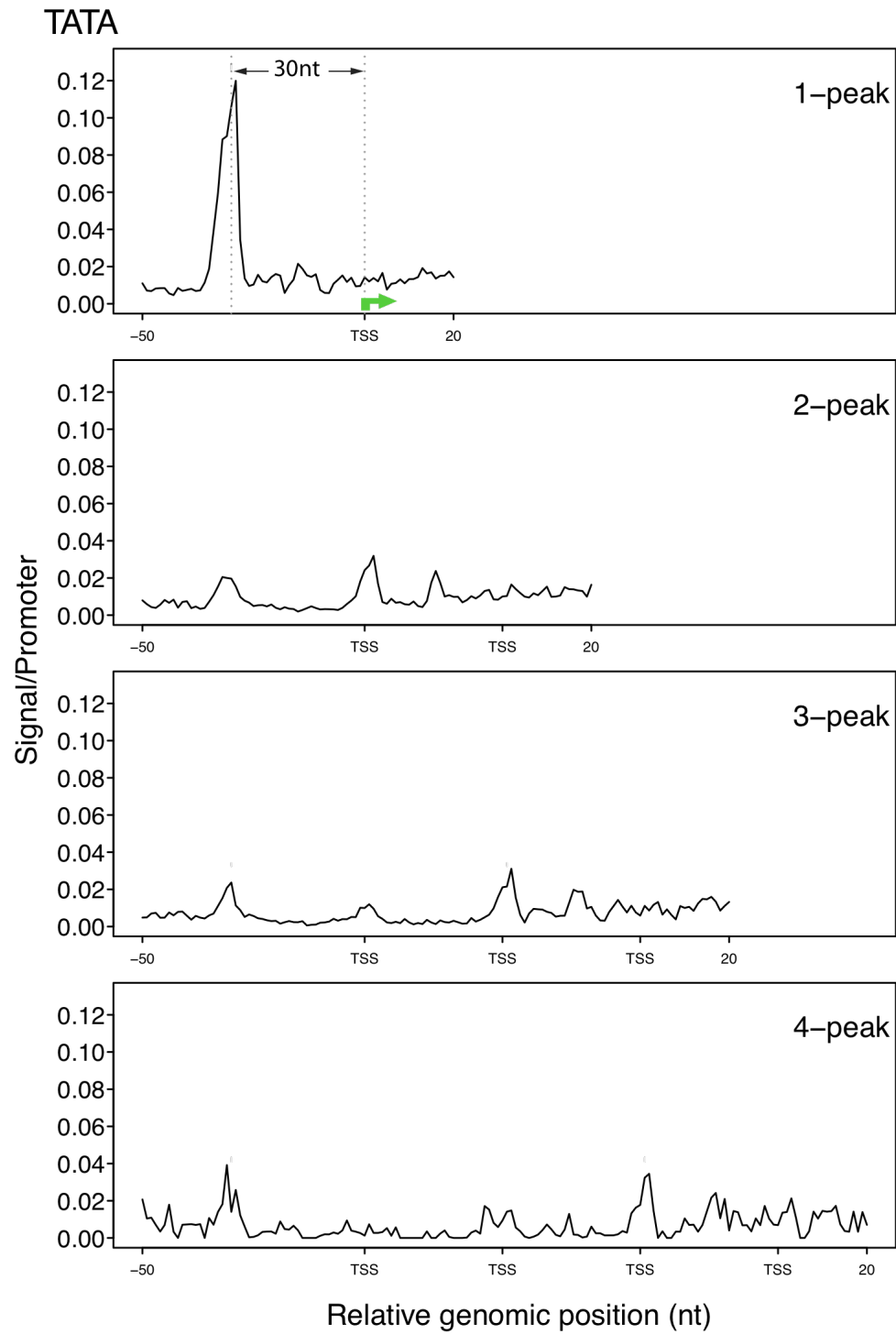

Supplement: Figure S7 — TATA motifs of subclasses of “scattered” TSSDs. Density plot of TATA-box extend −50 nt at 5′ of the first peak and 20 nt at 5′ of the last peak. The X-axis shows the genomic position relative to the peaks (TSS, indicated by a green arrow). The Y-axis shows the number of predicted sites per TSSD, as in Figure 3B. TATA motifs are dominant at around −30 nt in the 1-peak “scattered” TSSDs (top panel) while are strongly weakened in other subclasses. For each subclass, the TSSDs are aligned at their identified peaks, with the distance between two adjacent peaks rescaled to same width. (PDF) [file pone.0023409.s014.pdf]

**Figure S8. Alignments of pseudo and transcribed genes promoters**

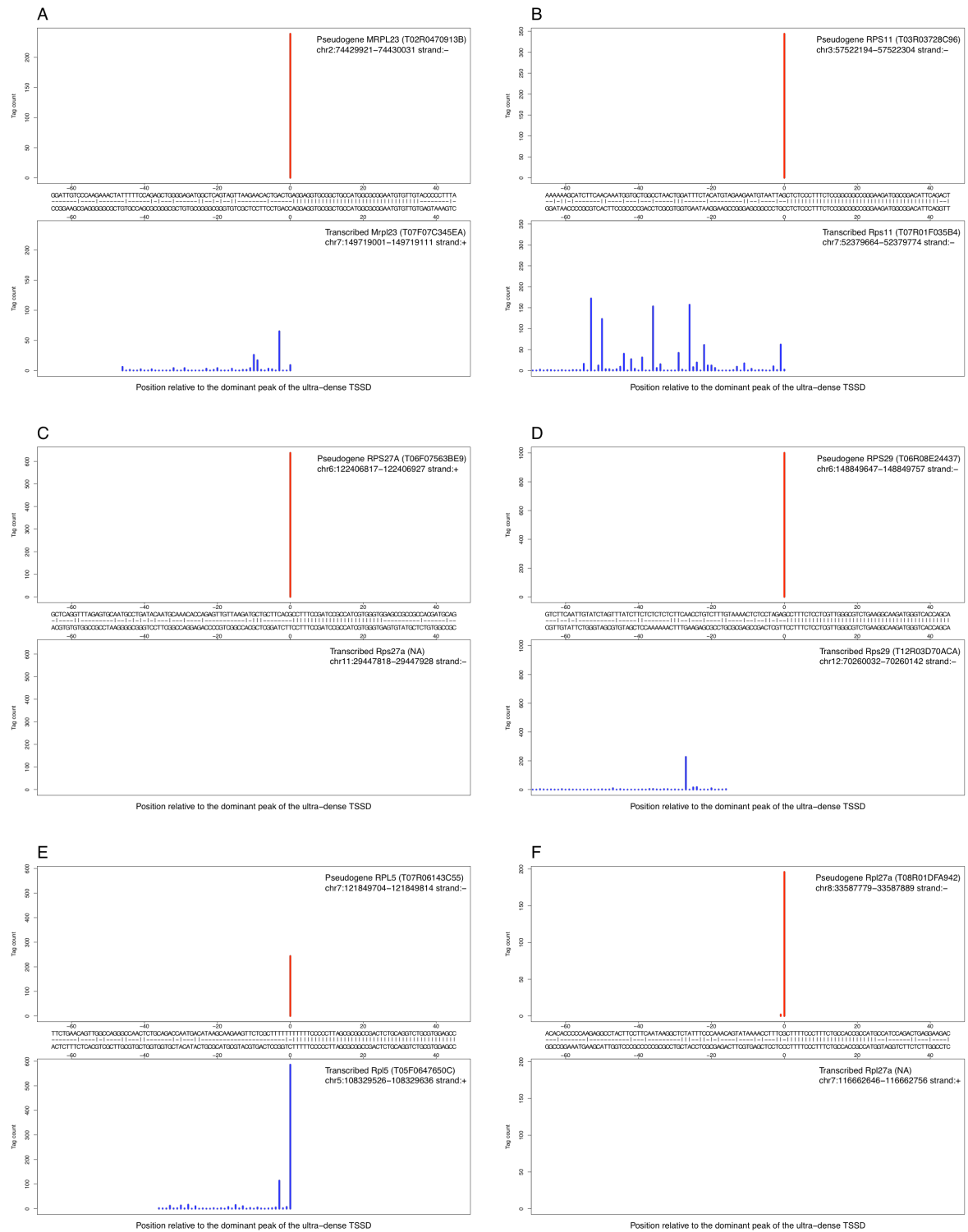

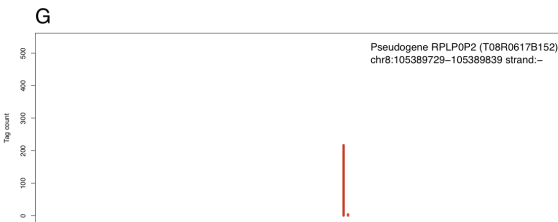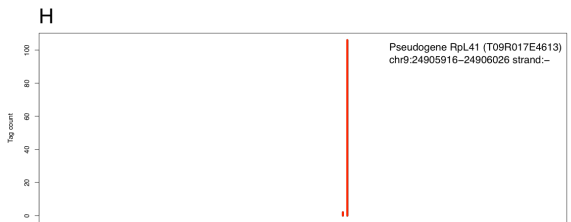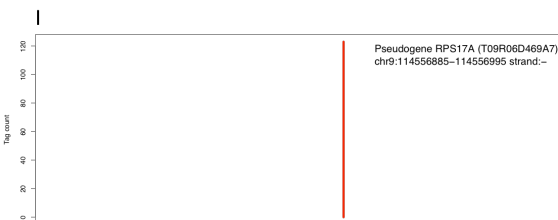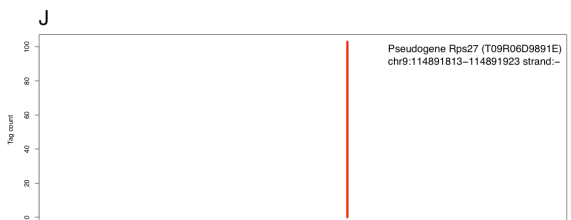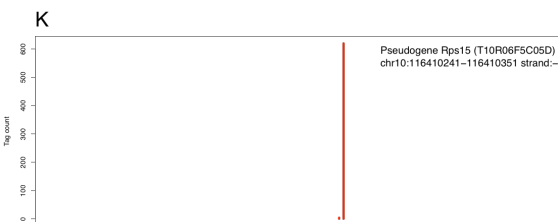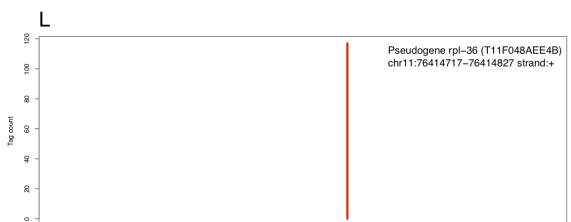

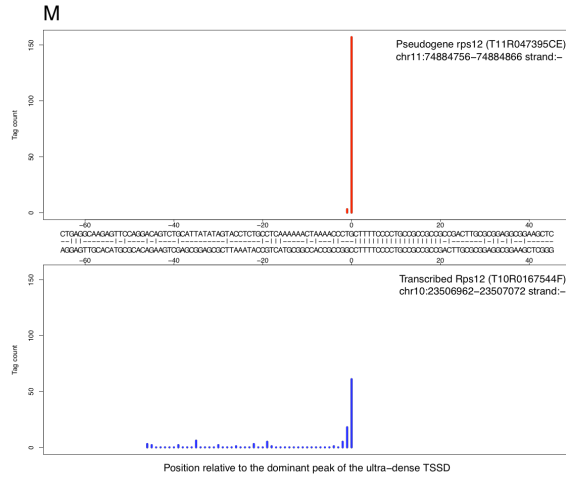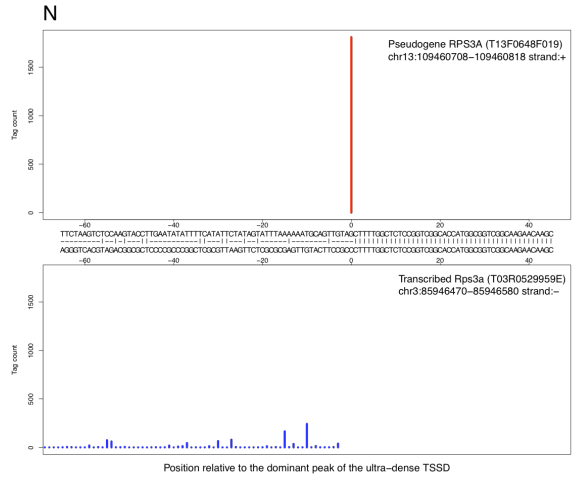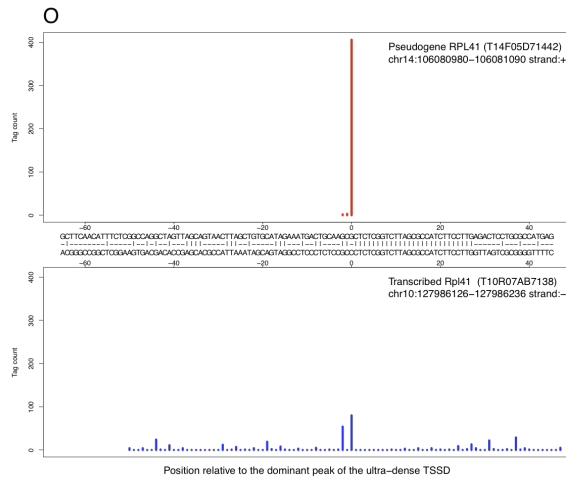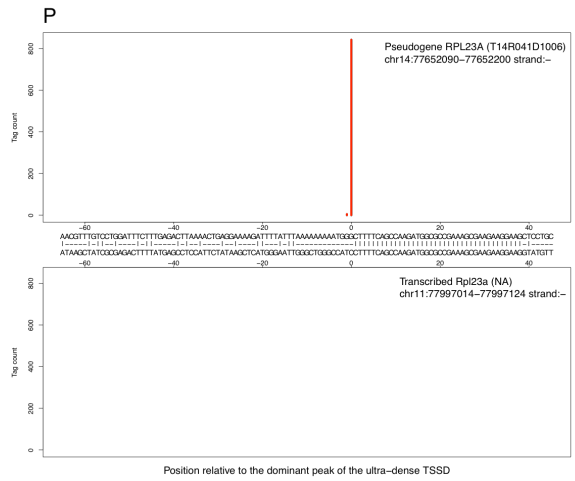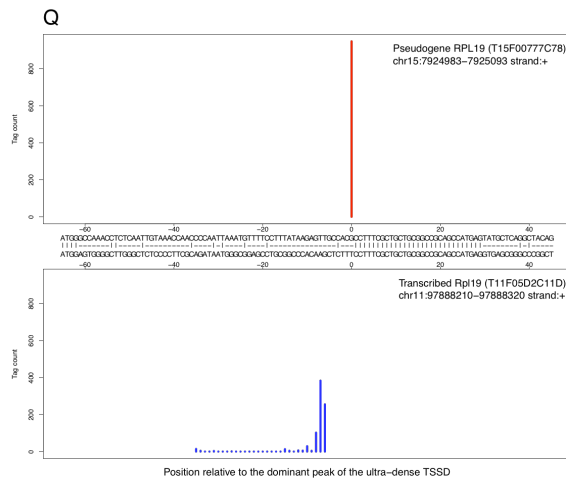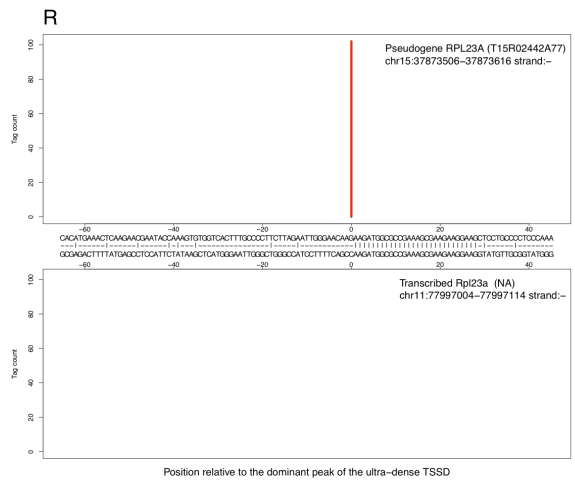

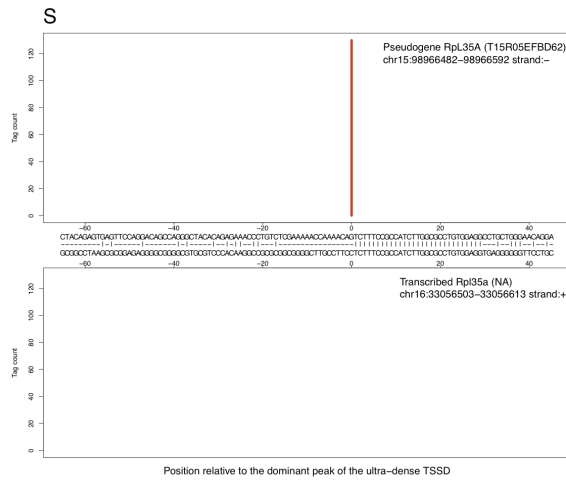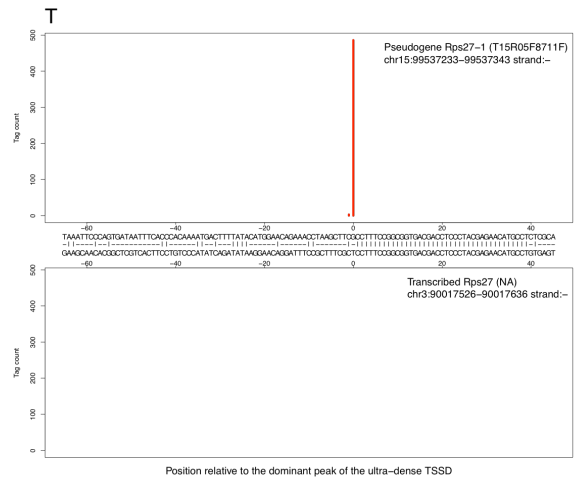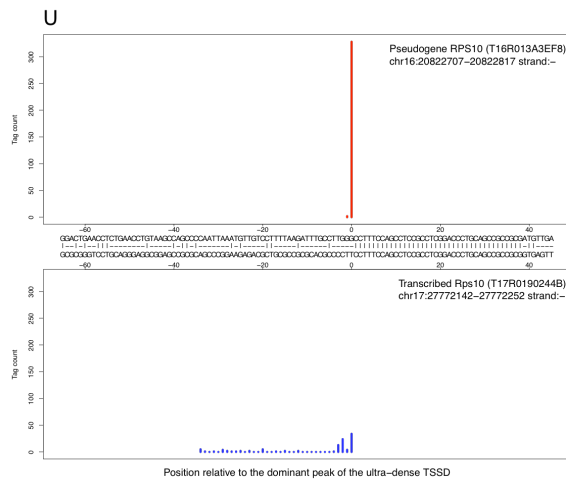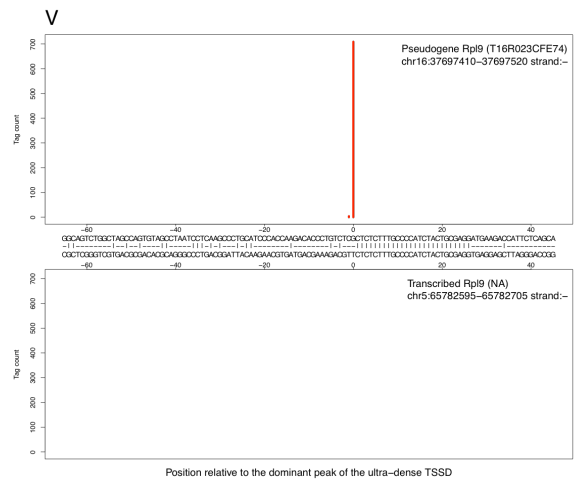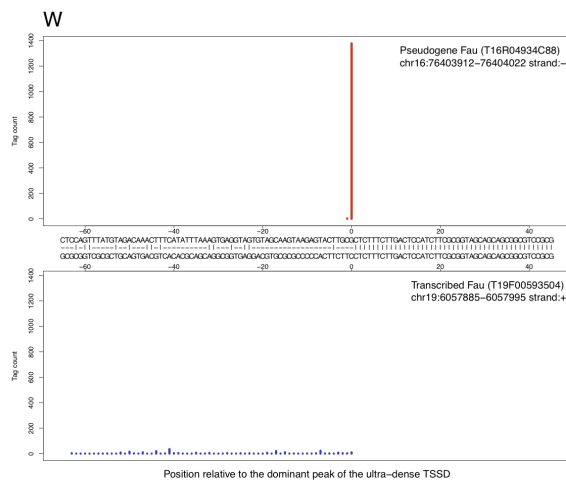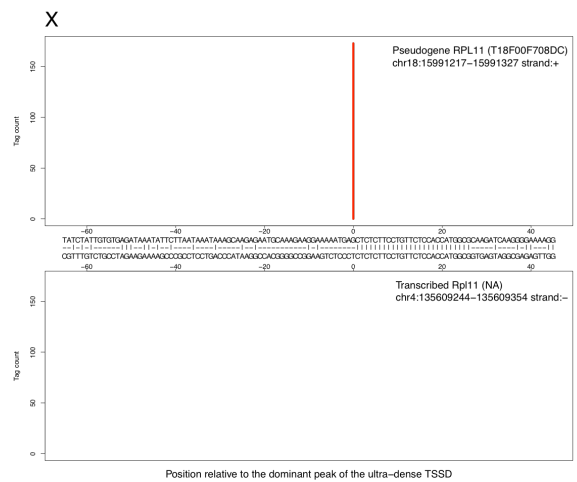

Y

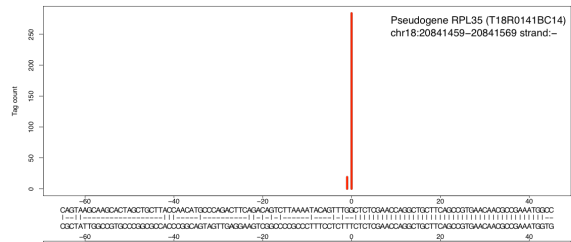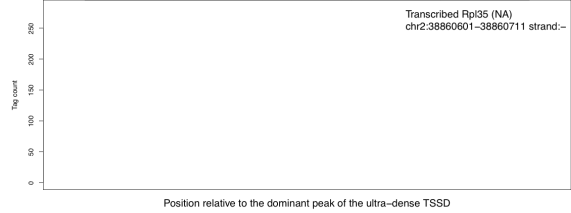

Supplement: Figure S8 — Alignments of pseudo and transcribed genes promoters. (A)–(Y) Alignments between pseudogene promoters (top) and the corresponding transcribed ribosomal protein gene promoters (bottom), as in Figure 5. Their sequences are aligned along the X-axis in between. X-axis shows the tag 5′ end position relative to the dominant peak (at position 0) of the pseudogene TSSDs; Y-axis shows the count of 5′ ends of the tags. (PDF) [file pone.0023409.s015.pdf]
